# Firing rate distributions in plastic networks of spiking neurons

**DOI:** 10.1162/netn_a_00442

**Published:** 2025-03-20

**Authors:** Marina Vegué, Antoine Allard, Patrick Desrosiers

**Affiliations:** Departament de Matemàtiques, Universitat Politècnica de Catalunya, Barcelona, Spain; Département de Physique, de Génie Physique et d’Optique, Université Laval, Québec, Canada; Centre Interdisciplinaire en Modélisation Mathématique, Université Laval, Québec, Canada; CERVO Brain Research Center, Québec, Canada

**Keywords:** Neural networks, Structural heterogeneity, Synaptic plasticity, Mean-field approach, Leaky integrate-and-fire neurons

## Abstract

In recurrent networks of leaky integrate-and-fire neurons, the mean-field theory has been instrumental in capturing the statistical properties of neuronal activity, like firing rate distributions. This theory has been applied to networks with either homogeneous synaptic weights and heterogeneous connections per neuron or vice versa. Our work expands mean-field models to include networks with both types of structural heterogeneity simultaneously, particularly focusing on those with synapses that undergo plastic changes. The model introduces a spike trace for each neuron, a variable that rises with neuron spikes and decays without activity, influenced by a degradation rate *r*_*p*_ and the neuron’s firing rate *ν*. When the ratio *α* = *ν*/*r*_*p*_ is significantly high, this trace effectively estimates the neuron’s firing rate, allowing synaptic weights at equilibrium to be determined by the firing rates of connected neurons. This relationship is incorporated into our mean-field formalism, providing exact solutions for firing rate and synaptic weight distributions at equilibrium in the high *α* regime. However, the model remains accurate within a practical range of degradation rates, as demonstrated through simulations with networks of excitatory and inhibitory neurons. This approach sheds light on how plasticity modulates both activity and structure within neuronal networks, offering insights into their complex behavior.

## Author Summary

Networks of spiking neurons are complex systems where the structure of connections and the activity patterns generated are deeply intertwined, a relationship often studied using mathematical approaches like the mean-field theory. However, previous studies have primarily focused on networks with limited structural variability, where either the connection strength is nearly identical across the network or the number of connections varies little from one neuron to another. This work takes a step forward by combining both types of structural variability and allowing connection strengths to adapt over time, thereby providing an extended mean-field theory. We derive exact solutions for the distribution of spiking rates and connection strengths at equilibrium and demonstrate their accuracy through numerical simulations, even beyond the defining parameter ranges, offering a more comprehensive and realistic perspective on the interplay between activity and structure in neuronal networks.

## INTRODUCTION

Synthetic networks of spiking neurons have been widely used to model the spontaneous activity of neuronal assemblies (Akil, Rosenbaum, & Josić, 2021; Amit & Brunel, 1997a, 1997b; Angulo-Garcia, Luccioli, Olmi, & Torcini, 2017; Cimeša, Ciric, & Ostojic, 2023; Duchet, Bick, & Byrne, 2023; Fusi & Mattia, 1999; Galán, 2008; Gast, Knösche, & Schmidt, 2021; Hartmann, Lazar, Nessler, & Triesch, 2015; Hennequin, Vogels, & Gerstner, 2012; Hutt, Mierau, & Lefebvre, 2016; Koch Ocker, 2023; La Camera, 2022; Lonardoni et al., 2017; Luccioli, Angulo-Garcia, & Torcini, 2019; Ostojic, 2014; Painchaud, Doyon, & Desrosiers, 2022; Pena, Zaks, & Roque, 2018; Sanzeni, Histed, & Brunel, 2022; Tartaglia & Brunel, 2017; Vogels & Abbott, 2005; Zillmer, Brunel, & Hansel, 2009). A common way to model the spiking activity of individual neurons is by means of the so-called *leaky **integrate-and-fire*** (LIF) model (Brunel & van Rossum, 2007; Gerstner, 2002). Despite being simple compared with more detailed spiking models (Izhikevich, 2004), the LIF model can reproduce some of the features observed in real neuronal assemblies when implemented on synthetic networks. For example, LIF neurons that receive both excitatory (E) and inhibitory (I) inputs that approximately compensate each other exhibit **spike trains** that are irregular and compatible with Poisson statistics, in agreement with the spontaneous activity measured experimentally (Shadlen & Newsome, 1994). In network models in which the average E input is compensated by the average I input for all neurons, such a balance can be maintained by the network dynamics because a temporary increase in the E activity rapidly induces an increase in the I activity (and vice versa) due to the recurrent connectivity (Renart et al., 2010; van Vreeswijk & Sompolinsky, 1996).

A clear advantage of the LIF model is that it is amenable to an analytical treatment: The **mean-field theory** of LIF and balanced neuronal networks can be used to describe the spiking statistics in the **stationary state** (Brunel, 2000). This theory takes into account not only the mean synaptic input received by individual neurons but also the input fluctuations caused by the irregularity of the spiking process in the presynaptic neurons. In a balanced state, in which the total mean synaptic input is close to zero, the spike emission is mainly driven by these input fluctuations. From the pioneering work of Amit and Brunel (1997a, 1997b), such mean-field formalisms have been used to predict the mean **firing rate** and firing rate distributions in networks of LIF neurons with various degrees of structural heterogeneity, including networks whose connectivity structure does not change in time (nonplastic) and networks whose synaptic efficacies have been modified by some plasticity mechanism. The term *structural heterogeneity* here refers either to a neuron-to-neuron variability in the number of inputs received from the network (i.e., the *in-degree*) or to a synapse-to-synapse variability in the synaptic efficacy (i.e., the *synaptic weight*).

Previous work has dealt with these two sources of structural heterogeneity separately. On the one hand, mean-field theory has been applied to nonplastic networks with homogeneous E/I synaptic weights and whose in-degrees are either homogeneous over different neurons (Brunel, 2000), slightly heterogeneous (as in networks with Erdős-Rényi connectivity) (Roxin, Brunel, Hansel, Mongillo, & van Vreeswijk, 2011), or determined by an arbitrary joint **in-/out-degree distribution** (Vegué & Roxin, 2019). In the latter case, the joint degree distribution can include correlations between individual in- and out-degrees, and this was shown to have an important influence on the resulting stationary distribution of firing rates (Vegué & Roxin, 2019). On the other hand, Amit and Brunel studied the case of networks with fixed in-degrees in which E/I synaptic weights are independently drawn from prescribed distributions, including networks whose weight distributions have been previously shaped by a learning process in which different subsets of neurons have been selected to store a set of activity patterns (Amit & Brunel, 1997b).

Together, these contributions (among several others) highlight the fact that any structural heterogeneity (be it a heterogeneity of in-degrees or of synaptic weights) causes a heterogeneity of firing rates in the stationary state and that this should be taken into account by the mean-field equations (Roxin et al., 2011; Vegué & Roxin, 2019). However, when the in-degrees are the same for all neurons, the heterogeneity of synaptic weights can be neglected under some conditions, and this greatly simplifies the mean-field equations and their steady-state solution (Brunel, 2000).

The aim of the present work is to study a rather general scenario in which both sources of structural heterogeneity (the one relative to the degree distribution and that of the weight distribution) are simultaneously taken into account. We assume that the connectivity structure (“who connects to whom”) is determined by a time-invariant “scaffold” on top of which the actual efficacy of every particular connection is defined by the synaptic weight, which can be stable in time or plastic. Hence, we consider networks without **structural plasticity** that can, however, exhibit **synaptic plasticity** (Caroni, Donato, & Muller, 2012; Knoblauch, Palm, & Sommer, 2010). The connectivity scaffold is determined by a joint in-/out-degree distribution but is fully random otherwise (i.e., configuration model; Cooper & Frieze, 2004; Newman, 2003). We examine synaptic weights under two distinct conditions. In the first scenario, the weights remain unchanged over time yet exhibit variability among the different synaptic connections. Each weight is independent and conforms to a predetermined distribution (model A). In the second scenario, we explore a dynamic setting where synaptic weights evolve over time, adjusting in response to neuronal activity according to a specific plasticity rule (model B).

As it is common in models of **spike-timing-dependent plasticity (STDP)**, the plasticity in our model is mediated by the introduction of spike traces (Akil et al., 2021; Chen & Jasnow, 2010; di Volo, Burioni, Casartelli, Livi, & Vezzani, 2014; Gerstner, Kistler, Naud, & Paninski, 2014; Morrison, Diesmann, & Gerstner, 2008; Pfister & Gerstner, 2006; Wang & Aljadeff, 2022). A trace associated to one neuron is a variable that increases by a fixed amount every time the neuron emits a spike and decays over time in the absence of neuronal activity. It may represent a chemical signal that is released in response to firing (Pfister & Gerstner, 2006). Its characteristic degradation rate is a measure of how fast the “memory” about the neuron’s spiking history is lost. In this work, we consider a single trace per neuron, in contrast with the usual implementation of plasticity rules based on spike traces where every neuron’s spikes contribute to different traces that differ in their degradation rate (i.e., two traces per neuron in pair-based STDP rules; Morrison et al., 2008; and three or more in triplet models; Pfister & Gerstner, 2006).

If the firing process is stochastic and controlled by an intrinsic firing rate *ν*, the trace itself is a stochastic variable whose temporal evolution depends on *ν* and on the trace’s degradation rate, *r*_*p*_. We perform a mathematical analysis of the trace, assuming that the spiking process is Poisson and the firing rate is constant in time. We show that the trace’s stationary probability density can be analytically computed and, in particular, (a) the trace can be rescaled so that it equals the firing rate *on average* and (b) the fluctuations around this mean vanish in the limit in which the ratio *α* = *ν*/*r*_*p*_ goes to infinity. Close to this limit, the trace can be used to estimate the underlying firing rate with high accuracy (we say that the trace is *reliable*).

In our plasticity model, the temporal evolution of each synaptic weight follows an ordinary differential equation (ODE) that depends on the weight itself and on the pre- and postsynaptic traces. Close to the limit of reliable traces, the synaptic weight at equilibrium can be thus expressed as a function of the firing rates underlying the pre- and postsynaptic spiking processes. This is the key ingredient that allows us to link the microscopic description of the neuronal activity (in terms of voltages, spikes, and traces) with its macroscopic mean-field description at equilibrium (in terms of firing rates).

We extend previous mean-field formalisms (Amit & Brunel, 1997a, 1997b; Brunel, 2000; Roxin et al., 2011; Vegué & Roxin, 2019) to networks with the two sources of structural heterogeneity described earlier, with and without plasticity (Models A and B, respectively). The solution to the mean-field equations allows the reconstruction of the firing rate distribution (and the synaptic weight distribution in model B) at equilibrium. This is done by invoking the central limit theorem (CTL), which allows to jointly regard the mean and the variance of the input to a given neuron, that a priori depend upon a whole unknown rate (and weight in Model B) probability distribution, as a Gaussian random vector that depends on a limited number of unknown statistics. These unknowns are computed by solving the mean-field equations, which specify the dependence of the unknowns on the unknowns themselves due to the fact that the network is recurrent, and, thus, the input and the output statistics are linked. The equations are exact in the limit of reliable traces, but they already provide accurate results when the degradation time constant of the trace, *τ_p_* = 1/*r*_*p*_, is of the order of seconds. This is shown by comparing the analytically computed rate/weight distributions with those obtained from simulating the whole process on a network.

## THE NEURONAL NETWORK MODEL

### The Neuronal Dynamics

We consider a network of *N* LIF neurons. The membrane voltage *V*_*i*_ of a neuron *i* in the network evolves in time according to

$$\frac{d}{dt}V_{i}\left(t\right)=-\frac{1}{\tau }V_{i}\left(t\right)+I_{i}\left(t\right),$$ (1)

where *τ* is a time constant and *I*_*i*_(*t*) is the input current received from other neurons. Whenever *V*_*i*_ reaches a threshold *V_θ_*, the neuron fires a spike and the voltage is reset to *V*_*r*_. During a period *τ_r_* immediately after firing, the neuron is refractory: Its voltage is fixed at *V*_*r*_ and the neuron cannot respond to the stimulation received from other neurons.

The input *I*_*i*_ is modeled as a sum of Dirac delta functions centered at the spike times of the neurons presynaptic to neuron *i* (plus a synaptic delay). We split this input into a recurrent input coming from the network itself ($I_{i}^{rec}$) and an input coming from a pool of external neurons ($I_{i}^{\mathrm{ext}}$):

$$I_{i}\left(t\right)=I_{i}^{rec}\left(t\right)+I_{i}^{\mathrm{ext}}\left(t\right),$$ (2a)

$$I_{i}^{rec}\left(t\right)=\sum_{j=1}^{N}a_{ij}\;w_{ij}\left(t\right)\sum_{k}\delta \left(t-t_{j}^{k}-d_{j}\right),$$ (2b)

$$I_{i}^{\mathrm{ext}}\left(t\right)=w_{\mathrm{ext}}\sum_{j=1}^{K_{\mathrm{ext}}}\sum_{k}\delta \left(t-t_{ij}^{k}\right).$$ (2c)

The first sum in Equation 2b runs over the neurons’ indices. The second sum runs over the spikes emitted in the past by neuron *j*, and $t_{j}^{k}$ denotes the *k*th spike time of neuron *j*. The delay in spike transmission is a parameter associated to neuron *j*, and it is given by *d*_*j*_. The binary matrix $A={\left(a_{ij}\right)}_{i,j=1}^{N}$ and the weighted matrix $W\left(t\right)={\left(w_{ij}\left(t\right)\right)}_{i,j=1}^{N}$ specify the connectivity in the network. The term *a*_*ij*_ is 1 whenever a connection from neuron *j* to neuron *i* exists, and is 0 otherwise. When *a*_*ij*_ = 1, *w*_*ij*_(*t*) gives the synaptic weight of the connection from *j* to *i* at time *t*. Equations 1, 2a, and 2b thus state that, whenever *a*_*ij*_ = 1, a spike emitted by the presynaptic neuron *j* at time $t_{j}^{k}$ will have an effect on neuron *i* at time $t_{j}^{k}+d_{j}$, and the effect is to make the postsynaptic voltage *V*_*i*_ jump a magnitude equal to the synaptic weight at this time, $w_{ij}\left(t_{j}^{k}+d_{j}\right)$.

The external input defined by Equation 2c has the same structure, but we assume that it is generated from a pool of *K*_ext_ external neurons that is unique for each postsynaptic neuron *i*. The time $t_{ij}^{k}$ is the arrival time of the *k*th spike emitted by the *j*th external neuron to neuron *i*. The synaptic weights from the external pool are assumed to be all the same for the sake of simplicity. While the spikes within the neuronal network are generated from the neurons’ voltages crossing the threshold, the external spikes are assumed to come from independent Poisson processes with a fixed rate *ν*_ext_.

### The Connectivity Structure

We call ***A*** the *binary adjacency matrix* and ***W***(*t*) the *weight matrix*. Matrix ***A*** is fixed (i.e., time independent) so it acts as a structural scaffold that determines which neurons can be connected. It also determines the (nonweighted) in- and out-degree of every neuron *i* via

$$K_{i}^{\text{in}}=\sum_{j=1}^{N}a_{ij},\;\;K_{i}^{\text{out}}=\sum_{j=1}^{N}a_{ji}.$$ (3)

We assume that ***A*** is a random instantiation from an ensemble of possible binary adjacency matrices. The ensemble is characterized by a joint in-/out-degree probability density function (p.d.f.) *ρ*_in, out_ in the following sense: The set of degree pairs ${\left\{\left(K_{i}^{\text{in}},K_{i}^{\text{out}}\right)\right\}}_{i=1}^{N}$ is a sample of *N* independent instantiations of a two-dimensional random vector that is distributed according to *ρ*_in, out_.

**Figure 1.**
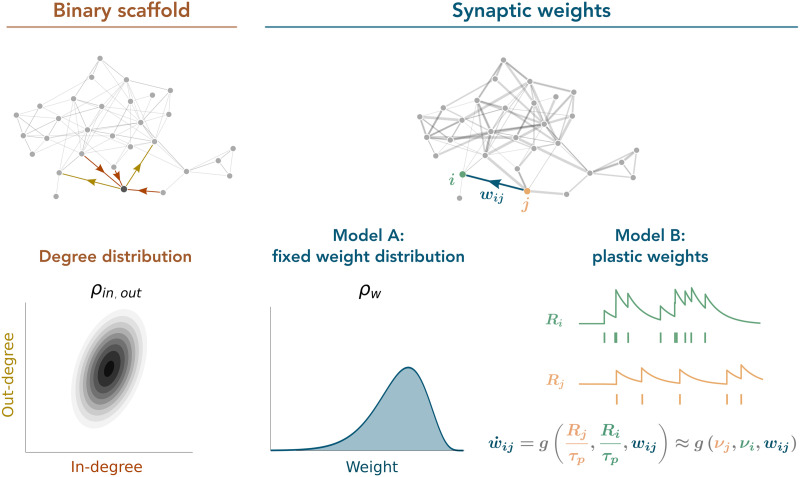
Schematics of the network structure. In both models, the network is built on a scaffold that is fixed and determines which neurons can be connected. The scaffold is such that individual in- and out-degrees follow a prescribed joint distribution defined by a density *ρ*_in,out_. On top of this scaffold, the synaptic weights vary from one synapse to the next. In model A, weights are fixed in time, and every weight is an independent realization of a common random variable following a prescribed distribution with density *ρ*_*w*_. In model B, every weight is plastic, and it varies in time as a function of the spiking traces of the pre- and postsynaptic neurons involved in the connection.

Contrary to ***A***, matrix ***W***(*t*) may change in time depending on pre- and postsynaptic neuronal activity. We explore the following two models for ***W***(*t*) (see Figure 1):

1. Model A. The structure of ***W*** is fixed in time: ***W***(*t*) = ***W***. Each synaptic weight *w*_*ij*_ is generated independently of the others from a common arbitrary weight probability distribution.
2. Model B. The matrix ***W***(*t*) is plastic: Each weight *w*_*ij*_(*t*) changes in time as a function of the activity of the pre- and postsynaptic neurons *j* and *i*. The details of the plasticity mechanism are given in the next section.

These models can be easily extended to networks with more than a single neuronal type or population (e.g., E and I neurons). For the sake of clarity, the mathematical analysis presented in the main text corresponds to a network with a single population, but we show and discuss results on networks with two populations in some of the main text’s figures. We provide the full mathematical analysis of the extended models in Supporting Information Sections S3 and S5.

### The Plasticity Rule

In Model B, for every pair (*i, j*) of connected neurons, the weight *w*_*ij*_ from *j* to *i* evolves in time as a function of the activities of neurons *i* and *j*. As it is standard in models of STDP (Gerstner et al., 2014; Morrison et al., 2008), besides membrane voltage, each neuron has associated a *spike trace* (also called *local variable* in the literature). This trace is a time-dependent variable that is a record of the neuron’s spiking activity. In particular, the trace *R*_*i*_ of neuron *i* exponentially decays with a characteristic time constant *τ_p_* and makes jumps of magnitude 1 every time the neuron fires a spike:

$$\frac{d}{dt}R_{i}\left(t\right)=-\frac{1}{\tau_{p}}R_{i}\left(t\right)+\sum_{k}\delta \left(t-t_{i}^{k}\right),$$ (4)

where, as in Equation 2b, *δ* is the Dirac delta function and ${\left\{t_{i}^{k}\right\}}_{k}$ is the collection of spike times of neuron *i*.

The trace *R*_*i*_ represents the concentration of a chemical signal that is released every time neuron *i* fires and that is degraded over time at a rate *r*_*p*_ = 1/*τ_p_*. This chemical signal could correspond to glutamate bound to its receptors, intracellular calcium, and second messengers, among many others (Pfister & Gerstner, 2006). The constant *τ_p_* acts as a “memory” parameter: It determines how much the spikes emitted in the past still have an impact on the trace at the present moment. A large *τ_p_* implies that the trace degradation is slow so the spiking memory is large, and vice versa. In the limit *τ_p_* → ∞, there is no trace degradation and *R*_*i*_(*t*) simply counts the total number of spikes emitted up to time *t* (assuming that *R*_*i*_ was 0 at time 0). Note that while many models of STDP assume the presence of two or more traces per neuron with distinct characteristic time constants (Morrison et al., 2008; Pfister & Gerstner, 2006), here, we assume a single trace per neuron.

As we show in detail in Supporting Information Section S1, if neuron *i* fires approximately as a Poisson process with a characteristic firing rate *ν_i_*, then its trace is a random process that can be used to approximate *ν_i_*. For this, the trace has to be normalized by *τ_p_*,

$${\hat{\nu }}_{i}\left(t\right)≔\frac{R_{i}\left(t\right)}{\tau_{p}},$$ (5)

so that, at equilibrium (i.e., when the probability distribution of *R*_*i*_(*t*) is independent of *t*), ${\hat{\nu }}_{i}\left(t\right)$ equals the firing rate *on average*:

$$E\left[{\hat{\nu }}_{i}\left(t\right)\right]=\nu_{i}.$$ (6)

We call ${\hat{\nu }}_{i}\left(t\right)$ the *normalized trace* or the *approximate firing rate* of neuron *i*. We will analyze the statistical properties of the normalized trace in The spike trace section.

In our plasticity model (Model B), the variation of the synaptic weight at time *t* depends on the value of the weight at time *t* and also on the value of the traces associated to the pre- and postsynaptic neurons involved in the connection at time *t*. Note that this makes our rule simpler than typical STDP rules, in which the instantaneous weight variation at time *t* depends not only on the value of the traces at *t* but also on the presence or absence of a pre/postspike at time *t* (see, e.g., Gerstner et al., 2014). Using the normalized trace defined by Equation 5, we express the instantaneous rate of change of a weight as a function of the weight itself and the pre- and postsynaptic normalized traces:

$$\frac{d}{dt}w_{ij}\left(t\right)=g\left({\hat{\nu }}_{j}\left(t\right),{\hat{\nu }}_{i}\left(t\right),w_{ij}\left(t\right)\right).$$ (7)

This relationship mirrors the classical equations for modeling synaptic plasticity based on neuronal firing rates while adhering to the principle of locality (Gerstner & Kistler, 2002).

For the derivation of our mean-field equations, we assume the function *g* to be such that in the steady-state solution of Equation 7; the weight *w*_*ij*_ is expressed as a sum of *n* ≥ 1 multiplicative functions of the traces, that is,

$$w_{ij}*=\sum_{k=1}^{n}g_{k}^{pre}\left({\hat{\nu }}_{j}^{*}\right)\;g_{k}^{\text{post}}\left({\hat{\nu }}_{i}^{*}\right),$$ (8)

where ${\hat{\nu }}_{i}^{*},{\hat{\nu }}_{j}^{*},w_{ij}^{*}$ are such that $g\left({\hat{\nu }}_{j}^{*},{\hat{\nu }}_{i}^{*},w_{ij}^{*}\right)=0$, and $g_{k}^{pre}$ and $g_{k}^{\text{post}}$ are arbitrary functions for all *k*. Notice that if the relation between the weight and the traces at equilibrium takes the general form

$$w_{ij}^{*}=g_{*}\left({\hat{\nu }}_{j}^{*},{\hat{\nu }}_{i}^{*}\right),$$ (9)

and g_*_ is of class ${𝒞}^{n}$, then we can use the Taylor theorem to approximate g_*_ by a sum of *n* multiplicative functions of ${\hat{\nu }}_{j}^{*}$ and ${\hat{\nu }}_{i}^{*}$.

For simplicity, we will restrict ourselves to the *n* = 1 case. In all our results, we take the function *g* to be of the form

$$g\left({\hat{\nu }}_{pre},{\hat{\nu }}_{\text{post}},w\right)=g_{0}\;{\hat{\nu }}_{pre}{\hat{\nu }}_{\text{post}}-\left({\hat{\nu }}_{\text{post}}^{2}+\in \right)w$$ (10)

so that the steady-state solution of Equation 7 is

$$w*=g^{pre}\left({\hat{\nu }}_{pre}^{*}\right)\;g^{\text{post}}\left({\hat{\nu }}_{\text{post}}^{*}\right)$$ (11)

with

$$g^{pre}\left(\nu \right)=g_{0}\;\nu ,\;g^{\text{post}}\left(\nu \right)=\frac{\nu }{\nu^{2}+\in }.$$ (12)

The parameter *g*_0_ is taken to be positive so that the first term of Equation 10 defines a pure Hebbian rule because the weight increase is larger when the pre- and the postsynaptic rates are simultaneously high (Gerstner & Kistler, 2002; Zenke & Gerstner, 2017). The second parameter, *ε*, is assumed to be positive and close to zero, ensuring the contribution of −*εw* to the homeostasis of the plasticity (Zenke & Gerstner, 2017). When *g*_0_ = 1 and *ε* = 0, this rule corresponds to Oja’s (1982) plasticity rule if we replace the normalized traces by the corresponding firing rates.

As we pointed out before, the trace and its normalized counterpart are stochastic variables due to the stochastic nature of the spiking process. As we show later, there is, nonetheless, a parameter regime in which the fluctuations of the normalized trace around its average can be neglected. This is the regime for which we develop the mean-field formalism when the weights are plastic.

## MEAN-FIELD FORMALISM

We are interested in studying macroscopic properties of the system defined by Equations 1–2c when the structure of the synaptic weights is heterogeneous and possibly plastic (i.e., given by Models A and B). We use the term *macroscopic property* to denote a feature that statistically characterizes the neuronal ensemble, regardless of its microscopic details. For example, a microscopic description of the activity of a single neuron in the network is provided by its *spike train*, that is, the collection of times at which the neuron has spiked. However, a simpler and probably more meaningful measure of the neuron’s activity is provided by the average number of spikes the neuron has emitted per unit time, that is, its *firing rate*. At the network level, the distribution of firing rates in the stationary state is a macroscopic property of the neuronal ensemble that is informative of the overall activity level in the network as well as of how heterogeneous this activity is. We notice that the term *stationary* here refers to the fact that the firing rates are stable in time. Despite this *statistical* stability, the underlying stochastic neuronal network remains highly active, with individual neurons still firing irregularly and exhibiting fluctuations. In the case of plastic synaptic weights, we are also interested in determining what the distribution of these weights will be in the stationary state.

The mean-field theory of networks of LIF neurons with homogeneous degrees or homogeneous synaptic weights has been extensively studied (Amit & Brunel, 1997a, 1997b; Brunel, 2000; Roxin et al., 2011; Vegué & Roxin, 2019). The formalism that we present here is an extension of this theory in networks that are heterogeneous in terms of both degrees and synaptic weights. The assumptions for the system to be well described macroscopically by the mean-field theory prevail, namely:

1. individual neurons fire as Poisson processes, so that Equation 2b is treated as a nondeterministic equation and Equation 1 becomes a *stochastic* differential equation;
2. each of these Poisson processes is defined by its characteristic firing rate and they are independent once the firing rates are known;
3. the absolute value of every synaptic weight is small compared with the threshold *V*_*θ*_, and the total number of inputs received by each neuron is large.

For Condition 1 to be approximately fulfilled, it is enough that the expectation of the total input current’s integral over a time window of length *τ* (i.e., the quantity *μ_i_* defined later by Equation 25; see Supporting Information Section S2) be below threshold (Feng, 2003, Chapter 15). In this case, the spiking process is mainly driven by the input fluctuations and it is thus irregular. Condition 2 is fulfilled when the set of presynaptic neurons to a given neuron has a small overlap from one neuron to another. This is accomplished when the network structure is random and sparse (that is, when the in-degrees are small compared with the network’s size, $K_{i}^{\text{in}}\ll N$ for all *i*). Condition 3 depends on the degree distribution, on *w*_ext_ and on the weight distribution chosen or the plasticity rule in place. Note that, according to Conditions 2 and 3, the in-degrees are large in absolute terms but small compared with the network’s size. We assume that all these conditions are approximately fulfilled in our networks.

In the following sections, we derive the mean-field equations that allow to analytically predict the firing rate and synaptic weight distributions in the stationary state. We first provide a brief mathematical analysis of the spike trace and its normalized counterpart. To make it clearer and easier to follow, we start by analyzing the much simpler case of a network with statistically equivalent neurons. We move afterward to the heterogeneous scenarios defined by Models A and B.

### The Spike Trace

Let us focus on the spike trace of a given random neuron *i*. If the spike times were known, Equation 4 could be solved analytically, yielding the solution

$$R\left(t\right)=R\left(t_{0}\right)\;e^{-\frac{t-t_{0}}{\tau_{p}}}+\sum_{k}\theta \left(t-t^{k}\right)\;e^{-\frac{t-t^{k}}{\tau_{p}}},$$ (13)

where *θ* denotes the Heaviside step function and where we omitted the subscript *i* to simplify the notation in what follows. In the mean-field formalism, however, we treat the spike train as a stochastic process that is well approximated by a Poisson process. This transforms the trace Equation 4 into a stochastic differential equation whose solution is given by a time-dependent p.d.f., *ρ*(*r*, *t*). This function allows us to compute the probability that, at time *t*, the trace lies within a given interval [*r*, *r* + d*r*]:

$$P\left(R\left(t\right)\in \left[r,r+dr\right]\right)=\int_{r}^{r+dr}\rho \left(s,t\right)\;ds.$$ (14)

If the firing process that determines the trace jumps is a Poisson process of rate *ν*, the function *ρ*(*r*, *t*) obeys the partial differential equation

$$\tau_{p}\frac{\partial }{\partial t}\rho \left(r,t\right)=\left(1-\alpha \right)\rho \left(r,t\right)+r\frac{\partial }{\partial r}\rho \left(r,t\right)+\alpha \rho \left(r-1,t\right)$$ (15)

with

$$\alpha ≔\tau_{p}\nu$$ (16)

(see Supporting Information Section S1.1 for details). In particular, the stationary distribution of *r, ρ*(*r*), fulfills

$$r\rho^{\prime }\left(r\right)=\left(\alpha -1\right)\rho \left(r\right)-\alpha \rho \left(r-1\right).$$ (17)

From Equation 15, we can obtain a system of ODEs for the moments of *R*(*t*). We denote by 〈*R*〉(*t*) the expectation of *R*(*t*) and by 〈*R*_*n*_〉(*t*) the centered moment of order *n* ≥ 0 of *R*(*t*):

$$\begin{array}{ll} \langle R\rangle \left(t\right)≔ & \int_{-\infty }^{\infty }r\rho \left(r,t\right)\;dr\\ \langle R_{n}\rangle \left(t\right)≔ & \int_{-\infty }^{\infty }{\left[r-\langle R\rangle \left(t\right)\right]}^{n}\rho \left(r,t\right)\;dr\;\text{for}\;n\geq 0. \end{array}$$ (18)

Clearly, 〈*R*_0_〉(*t*) = 1 and 〈*R*_1_〉(*t*) = 0 for all *t*. The equations (see Supporting Information Section S1.2) are

$$\begin{array}{l} \tau_{p}\langle \dot{R}\rangle =-\langle R\rangle +\alpha \\ \tau_{p}\langle {\dot{R}}_{n}\rangle =-n\langle R_{n}\rangle +\alpha \left(1+\sum_{k=2}^{n-2}\left(\begin{array}{c} n\\ k \end{array}\right)\langle R_{k}\rangle \right)\;\text{for}\;n\geq 2. \end{array}$$ (19)

Note the similarity between the ODE for 〈*R*〉 and that of *R* itself, Equation 4: In the averaged version, the spike train $\sum_{k}\delta \left(t-t_{i}^{k}\right)$ has been replaced by the firing rate *ν* = *α*/*τ_p_*. We see that, *on average*, the variable *R* approaches exponentially to *α* = *τ_p_ν*, meaning that the rescaled stochastic variable

$$\hat{\nu }\left(t\right)≔R\left(t\right)/\tau_{p}$$ (20)

allows to approximately recover the firing rate *ν* from *R*. The equations for the expectation $\langle \hat{\nu }\rangle \left(t\right)$ and variance $\langle {\hat{\nu }}_{2}\rangle \left(t\right)$ of $\hat{\nu }\left(t\right)$ are

$$\begin{array}{lll} \tau_{p}\langle \dot{\hat{\nu }}\rangle & = & -\langle \hat{\nu }\rangle +\nu \\ \tau_{p}\langle {\dot{\hat{\nu }}}_{2}\rangle & = & -2\langle {\hat{\nu }}_{2}\rangle +\frac{\nu }{\tau_{p}}. \end{array}$$ (21)

**Figure 2.**
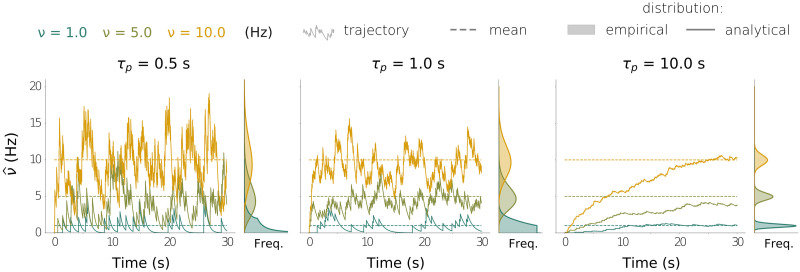
Trace-dependent estimated firing rate $\hat{\nu }$ as defined by Equations 4 and 20 for different values of the true firing rate *ν* and the memory constant *τ*_*p*_. In each plot, we show three examples of temporal trajectories for $\hat{\nu }\left(t\right)$ (*ν* = 1 Hz, *ν* = 5 Hz, and *ν* = 10 Hz), all of them starting at *t* = 0. The dashed lines indicate the predicted mean value at equilibrium, $\langle \hat{\nu }\rangle =\nu$. To the right of each plot, we show the p.d.f. of $\hat{\nu }$ at equilibrium, both analytical (obtained by numerically solving Equation 17 for the density of *R* and appropriately rescaling) and empirical (obtained by simulating an ensemble of 50,000 independent trajectories and computing the resulting histogram at time *t* = 5*τ*_*p*_).

From this, we derive several conclusions. First, the larger the memory constant *τ_p_*, the slower the convergence to the stationary distribution. Second, the coefficient of variation (standard deviation-to-mean ratio) for both *R* and $\hat{\nu }$ at equilibrium is

$$CV=\frac{1}{\sqrt{2\alpha }}=\frac{1}{\sqrt{2\tau_{p}\nu }},$$

so the estimation of *ν* through $\hat{\nu }$ becomes more accurate as the product of the memory constant and the true firing rate increases. In fact, in the limit *α* → ∞, *R* and $\hat{\nu }$ are normally distributed at equilibrium and *CV* = 0 (see Supporting Information Section S1.4). Conversely, for small *α*, the stationary distributions of *R* and $\hat{\nu }$ are highly non-Gaussian and they exhibit a large CV. Figure 2 shows typical trajectories and the stationary distribution of $\hat{\nu }$ for different values of *ν* and *τ_p_*.

As shown in this figure, the approximate rate $\hat{\nu }$ can be highly noisy, especially when the product *τ_p_ν* is small. When used to implement a plasticity rule, this variable can thus lead to highly varying synaptic weights, and this could make the firing rates vary accordingly. Thus, a true stationary state of the system, that is, a state in which both weights and rates remain stable in time, can only be reached in the limit of stable traces, that is, in the limit *τ_p_ν* → ∞. For finite values of *τ_p_ν*, the stationary state is only reached approximately. In what follows, we fix *τ_p_* to be large enough. The implication is that we can reasonably assume that the normalized trace $\hat{\nu }$ is a good approximation of the firing rate *ν* in the stationary state, that is,

$$\hat{\nu }\left(t\right)\approx \nu ,$$ (22)

so that we can rewrite the steady-state solution to the plasticity rule (Equation 11) as

$$w^{*}\approx g^{pre}\left(\nu_{pre}^{*}\right)\;g^{\text{post}}\left(\nu_{\text{post}}^{*}\right).$$ (23)

The biological interpretation of this assumption is that the trace is slowly degraded, so the memory of the trace on the spiking activity is large. This allows the synaptic weights to respond only to slow temporal variations of the firing rates.

### Introduction to the Mean-Field Formalism

From now on, we assume that our system is in a state characterized by a stationary distribution of firing rates, so in order to simplify the notation, we remove the asterisks (*) on the stationary weights and firing rates.

Under Conditions 1, 2, and 3, the stationary firing rate of a neuron *i* in the network is given by the equations (Brunel, 2000):

$$\begin{array}{l} \nu_{i}=\phi \left(\mu_{i},\sigma_{i}\right),\\ \phi {\left(\mu ,\sigma \right)}^{-1}=\tau_{r}+\tau \sqrt{\pi }\int_{\frac{V_{r}-\mu }{\sigma }}^{\frac{V_{\theta }-\mu }{\sigma }}\exp\left(u^{2}\right)\mathrm{erfc}\left(-u\right)\;du \end{array}$$ (24)

where *μ_i_* and *σ_i_* are, respectively, the mean and the standard deviation of the integral of the total input current *I*_*i*_(*t*) (Equation 2a) over a time window of length *τ*, that is,

$$\begin{array}{l} \mu_{i}=\tau \left(\sum_{j=1}^{N}a_{ij}w_{ij}\nu_{j}+K_{\mathrm{ext}}w_{\mathrm{ext}}\nu_{\mathrm{ext}}\right),\\ \sigma_{i}^{2}=\tau \left(\sum_{j=1}^{N}a_{ij}w_{ij}^{2}\nu_{j}+K_{\mathrm{ext}}w_{\mathrm{ext}}^{2}\nu_{\mathrm{ext}}\right), \end{array}$$ (25)

where *ν*_1_, …, *ν_N_* are the stationary firing rates of the neurons in the network (see Supporting Information Section 2 for details). Evaluating Equations 24 and 25 requires knowing what all the firing rates and all the synaptic weights are at equilibrium. However, the firing rate distribution (and the synaptic weight distribution in Model B) are not known a priori: They are the unknowns of our problem. The strategy to solve the problem is based on assuming that, whatever the rate and weight distributions are, the sums over *j* in Equation 25 are sums taken from *independent realizations of a common random vector* or realizations of weakly correlated random variables as in Rio (2017) and Rosenblatt (1956). This allows us to apply the CLT to these sums to conclude that they approximately follow a Gaussian distribution that is determined by a few statistical parameters. This step is key because it greatly reduces the space of the system’s unknowns from a whole distribution to a few parameters. The goal is then to compute these parameters. We will soon make these ideas more precise for the different model variations presented earlier.

### Network with Equivalent Neurons

We start by studying the simplest scenario: when all the neurons in the network are statistically identical. This occurs when the in-degrees are the same for all the neurons, and the synaptic weights are either the same for all the synapses (Model A) or evolve in time according to the same form of plasticity rule (Model B). This homogeneity results in a homogeneity of firing rates and synaptic weights in both models, so *the* problem’s unknown is the stationary firing rate *ν* (together with the stationary weight *w* in Model B).

We denote the in-degree of every neuron by *K*. The quantities *μ_i_* and *σ_i_* of Equation 25 in this case do not depend on *i* and reduce to

$$\begin{array}{lll} \mu =\tau \left(Kw\nu +K_{\mathrm{ext}}w_{\mathrm{ext}}\nu_{\mathrm{ext}}\right),\\ \sigma^{2}=\tau \left({Kw}^{2}\nu +K_{\mathrm{ext}}w_{\mathrm{ext}}^{2}\nu_{\mathrm{ext}}\right). & & \end{array}$$ (26)

If there is no plasticity in the network (Model A), meaning that all the synaptic weights are set to a known value *w*, then, according to Equation 24, the stationary firing rate *ν* is found by solving

$$\nu =\phi \left(\mu \left(\nu \right),\sigma \left(\nu \right)\right)$$ (27)

with

$$\begin{array}{l} \mu \left(\nu \right)=\tau \left(Kw\nu +K_{\mathrm{ext}}w_{\mathrm{ext}}\nu_{\mathrm{ext}}\right),\\ \sigma^{2}\left(\nu \right)=\tau \left({Kw}^{2}\nu +K_{\mathrm{ext}}w_{\mathrm{ext}}^{2}\nu_{\mathrm{ext}}\right). \end{array}$$ (28)

If there is a plasticity mechanism of the form described earlier (Model B), the value of all the synaptic weights in the stationary state is specified by the stationary firing rate through *w* = *w*(*ν*), so the equation to solve is Equation 27 with

$$\begin{array}{l} \mu \left(\nu \right)=\tau \left(Kw\left(\nu \right)\nu +K_{\mathrm{ext}}w_{\mathrm{ext}}\nu_{\mathrm{ext}}\right),\\ \sigma^{2}\left(\nu \right)=\tau (Kw{\left(\nu \right)}^{2}\nu +K_{\mathrm{ext}}w_{\mathrm{ext}}^{2}\nu_{\mathrm{ext}}). \end{array}$$ (29)

### Heterogeneous Network with No Plasticity (Model A)

We move now to the case of heterogeneous connectivity with synaptic weights that are constant in time. The binary connectivity structure is defined by a joint distribution of in-/out-degrees. The synaptic weights are generated independently from a known weight distribution.

As stated before, in the stationary state, the firing rate of a random neuron *i* can be computed through Equations 24 and 25. We can rewrite Equation 25 as

$$\begin{array}{l} \mu_{i}=\tau \left(S_{\mu }^{i}+K_{\mathrm{ext}}w_{\mathrm{ext}}\nu_{\mathrm{ext}}\right),\\ \sigma_{i}^{2}=\tau \left(S_{\sigma }^{i}+K_{\mathrm{ext}}w_{\mathrm{ext}}^{2}\nu_{\mathrm{ext}}\right), \end{array}$$ (30)

with

$$\begin{array}{l} S_{\mu }^{i}≔\sum_{j=1}^{N}a_{ij}w_{ij}\nu_{j},\\ S_{\sigma }^{i}≔\sum_{j=1}^{N}a_{ij}w_{ij}^{2}\nu_{j}. \end{array}$$ (31)

The key step is to deal with the sums of Equation 31. To simplify the notation, let us reorder the indices of the presynaptic neurons to neuron *i* so that these indices are now 1, …, *K*_*i*_, where *K*_*i*_ is the in-degree of neuron *i*, $K_{i}^{\text{in}}=K_{i}$. This allows us to rewrite Equation 31 as

$$S_{i}≔\left(\begin{array}{l} S_{\mu }^{i}\\ S_{\sigma }^{i} \end{array}\right)=\sum_{j=1}^{K_{i}}\left(\begin{array}{l} w_{ij}\nu_{j}\\ w_{ij}^{2}\nu_{j} \end{array}\right).$$ (32)

In the mean-field formulation, we treat the neurons statistically, so the in-degree *K*_*i*_ is a random variable taken from the in-degree distribution imposed in the network, and once *K*_*i*_ is known, the sum in Equation 34 can be assumed to be a sum over *K*_*i*_ independent and identically distributed (i.i.d.) random vectors $v_{1}^{i},\ldots ,v_{K_{i}}^{i}$,

$$v_{j}^{i}=\left(\begin{array}{l} w_{ij}\nu_{j}\\ w_{ij}^{2}\nu_{j} \end{array}\right).$$ (33)

The distribution of a presynaptic rate *ν_j_* does not depend on *K*_*i*_. This is ensured by the random connectivity structure in the network (beyond the degree distribution imposed), which makes the in-degrees of connected neurons be independent random variables (see Supporting Information Sections S3.3 and S3.4 for a proper justification). This would not be the case in an assortative network in which neurons with large in-degrees tend to be connected to neurons with large in-degrees. The independence between in-degrees of connected neurons implies that the firing rate of a presynaptic neuron (which is a function of its in-degree) is also independent of the postsynaptic in-degree.

Also, the postsynaptic firing rate can be assumed to be independent of a presynaptic rate *ν_j_* when the synaptic weight *w*_*ij*_ is small enough so that the influence of a single presynaptic neuron on a postsynaptic neuron is negligible. Because of all these reasons, the presynaptic rates *ν*_1_, …, *ν_K_i__* can be regarded as i.i.d. random variables whose distribution is independent of *i*.

Since the weights are also chosen independently from a common weight distribution, the result is that the vectors $v_{1}^{i},\ldots ,v_{K_{i}}^{i}$ are i.i.d. and the distribution that characterizes them is independent of the postsynaptic neuron *i*. This “universality” feature of the set of vectors ${\left\{v_{j}^{i}\right\}}_{i,j}$ is key in our mean-field calculation. Let

$$m=\left(\begin{array}{l} m_{\mu }\\ m_{\sigma } \end{array}\right),\;\Sigma =\left(\begin{array}{ll} s_{\mu }^{2} & c_{\mu \sigma }\\ c_{\mu \sigma } & s_{\sigma }^{2} \end{array}\right)$$ (34)

be the mean vector and the covariance matrix of $v_{j}^{i}$, that is,

$$\begin{array}{l} m_{\mu }≔E\left[\;w_{ij}\nu_{j}\;|\;j\to i\;\right],\\ s_{\mu }^{2}≔Var\left[\;w_{ij}\nu_{j}\;|\;j\to i\;\right],\\ m_{\sigma }≔E\left[\;w_{ij}^{2}\nu_{j}\;|\;j\to i\;\right],\\ s_{\sigma }^{2}≔Var\left[\;w_{ij}^{2}\nu_{j}\;|\;j\to i\;\right],\\ c_{\mu \sigma }≔Cov\left[\;w_{ij}\nu_{j},w_{ij}^{2}\nu_{j}\;|\;j\to i\;\right], \end{array}$$ (35)

where *j* → *i* indicates that *j* is a presynaptic neuron of *i*, that is, *a*_*ij*_ = 1. As it was pointed out in Vegué and Roxin (2019) (and it is explained in detail in Supporting Information Section S3.3), this condition cannot be neglected. A neuron that is presynaptic to another neuron tends to have a larger out-degree than a neuron picked at random (being presynaptic, in particular, means that your out-degree is at least 1). If individual in- and out-degrees in the network are correlated, the distribution of in-degrees among the presynaptic neurons is going to be biased compared with the distribution of in-degrees in the network. Since the firing rate depends on the in-degree, this, in turn, will bias the distribution of firing rates among the presynaptic neurons, and the statistical parameters in Equation 35 will be biased too. This bias can be precisely quantified as we will show later.

Once *K*_*i*_ is known, and if it is large enough, the multidimensional version of the CLT ensures that the vector ***S****_i_* will be approximately distributed as a bivariate normal vector with mean vector *K*_*i*_***m*** and covariance matrix *K*_*i*_Σ:

$$\begin{array}{lllll} S_{i} & = & \left(\begin{array}{c} S_{\mu }^{i}\\ S_{\sigma }^{i} \end{array}\right) & = & K_{i}\left(\begin{array}{c} m_{\mu }\\ m_{\sigma } \end{array}\right)+\sqrt{K_{i}}\left(\begin{array}{c} Y_{i}\\ Z_{i} \end{array}\right), \end{array}$$ (36)

where

$$\begin{array}{lllllll} \left(\begin{array}{c} Y_{i}\\ Z_{i} \end{array}\right) & ∼ & N\left(0,\Sigma \right). & & & & \end{array}$$ (37)

We denote by *m* and *s*^2^ the mean and variance of the rate of a presynaptic neuron, respectively:

$$\begin{array}{l} m≔E\left[\nu_{j}|\;j\;\text{is}\;a\;\text{presynaptic neuron}\right],\\ s^{2}≔Var\left[\nu_{j}|\;j\;\text{is}\;a\;\text{presynaptic neuron}\right]. \end{array}$$ (38)

Let ***θ*** = (*m*, *s*^2^). Since any synaptic weight is independent of the firing rate of its presynaptic neuron, the moments defined in Equation 35 are expressed as a function of the moments of the weight distribution and the pair of rate statistics ***θ*** by

$$\begin{array}{l} m_{\mu }\left(\theta \right)=E\left[w\right]\;m,\\ s_{\mu }^{2}\left(\theta \right)=E\left[w^{2}\right]\;s^{2}+Var\left[w\right]\;m^{2},\\ m_{\sigma }\left(\theta \right)=E\left[w^{2}\right]\;m,\\ s_{\sigma }^{2}\left(\theta \right)=E\left[w^{4}\right]\;s^{2}+Var\left[w^{2}\right]\;m^{2},\\ c_{\mu \sigma }\left(\theta \right)=E\left[w^{3}\right]\;s^{2}+\left(E\left[w^{3}\right]-E\left[w\right]\;E\left[w^{2}\right]\right)\;m^{2}. \end{array}$$ (39)

Thus, to compute the statistics $m_{\mu },s_{\mu }^{2},m_{\sigma },s_{\sigma }^{2}$ and *c_μσ_*, it is enough to know the mean and the variance of the rate distribution of presynaptic neurons, *m* and *s*^2^. Once these two parameters are known, the distribution of the vector ***S****_i_* is determined through Equations 34, 36, 37, and 39. The firing rate *ν_i_* of a neuron *i* in the network is therefore specified by the pair of *mean-field parameters, **θ*** = (*m*, *s*^2^), and by a triplet of *identity variables* associated to that neuron, ***X****_i_* = (*K*_*i*_, *Y*_*i*_, *Z*_*i*_) (whose distribution, in turn, depends on the mean-field parameters):

$$\nu_{i}=\nu \left(\theta ,X_{i}\right)=\phi \left(\mu \left(\theta ,X_{i}\right),\sigma \left(\theta ,X_{i}\right)\right),$$ (40a)

$$\begin{array}{l} \mu \left(\theta ,X_{i}\right)=\tau \left(K_{i}m_{\mu }\left(\theta \right)+\sqrt{K_{i}}Y_{i}+K_{\mathrm{ext}}w_{\mathrm{ext}}\nu_{\mathrm{ext}}\right)\\ \sigma 2\left(\theta ,X_{i}\right)=\tau \left(K_{i}m_{\sigma }\left(\theta \right)+\sqrt{K_{i}}Z_{i}+K_{\mathrm{ext}}w_{\mathrm{ext}}^{2}\nu_{\mathrm{ext}}\right). \end{array}$$ (40b)

If the neuron is randomly chosen in the network, its identity variables *K*_*i*_, *Y*_*i*_, *Z*_*i*_ are random variables: *K*_*i*_ is distributed according to the in-degree distribution imposed in the network, and (*Y*_*i*_, *Z*_*i*_) is a normal bivariate vector with zero mean and covariance matrix Σ = Σ(***θ***), as stated by Equation 37. The vector (*Y*_*i*_, *Z*_*i*_) is independent of *K*_*i*_, and the identity vectors of all the neurons, ***X***_1_, …, ***X****_N_*, are i.i.d. In sum, the whole rate distribution in the network can be reconstructed from only two rate statistics: *m* and *s*^2^.

The problem, then, reduces to computing these two statistics. This can be done by using their definitions as the mean and the variance of the rate of a random presynaptic neuron: They should fulfill

$$\begin{array}{l} m=\int_{0}^{\infty }\int_{-\infty }^{\infty }\int_{-\infty }^{\infty }\nu \left(\theta ,x\right)\rho_{X}^{\theta }\left(x\right)dx≕F_{m}\left(\theta \right),\\ s^{2}=\int_{0}^{\infty }\int_{-\infty }^{\infty }\int_{-\infty }^{\infty }{\left(\nu \left(\theta ,x\right)-m\right)}^{2}\rho_{X}^{\theta }\left(x\right)\;dx≕F_{s^{2}}\left(\theta \right), \end{array}$$ (41)

where ***x*** = (*k*, *y*, *z*) and $\rho_{X}^{\theta }\left(x\right)$ is the joint p.d.f. of the triplet ***X****_i_* = (*K*_*i*_, *Y*_*i*_, *Z*_*i*_) *for a presynaptic neuron*:

$$\rho_{X}^{\theta }\left(x\right)=\rho_{K}^{pre}\left(k\right)\;\rho_{Y,Z}^{\theta }\left(y,z\right),$$ (42)

with $\rho_{K}^{pre}$ being the p.d.f. of the in-degree of a presynaptic neuron (see Supporting Information Section S3.3 on how to compute it) and $\rho_{Y,Z}^{\theta }$ being the p.d.f. of a normal bivariate vector with mean **0** and covariance matrix Σ(***θ***).

For the system to be consistent, Equation 41 must be fulfilled. One should thus find the pair of mean-field parameters ***θ*** = (*m*, *s*^2^) by solving the system of two unknowns and two equations

$$\theta =F\left(\theta \right),$$ (43)

with *F*(***θ***) ≔ (*F*_*m*_, *F*_*s*^2^_)(***θ***) and *F*_*m*_, *F*_*s*^2^_ being the functions defined in Equation 41.

Once ***θ*** is known, the network’s firing rate distribution can be numerically reconstructed by creating a large sample of triplets {***X****_i_*}*_i_* and then using it to compute the corresponding sample of firing rates by applying Equation 40(a and b). Note that to create the triplet sample, we must use the in-degree distribution in the network, not the in-degree distribution among presynaptic neurons as in Equations 41 and 42.

**Figure 3.**
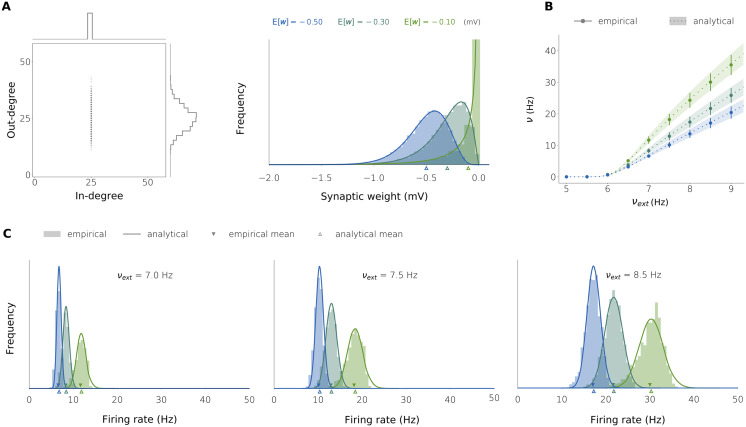
Firing rate distributions in three inhibitory networks with heterogeneous synaptic weights. (A) Details of the connectivity structure. Left: in-/out-degree histogram (the same in the three networks), with fixed in-degree and variable out-degree; right: synaptic weight histograms. The synaptic weights have been created independently with *w*_*ij*_ ∼ −Gamma(*κ*, *θ*) with mean $E\left[w\right]\in \left\{-0.1,-0.3,-0.5\right\}$ and variance Var[*w*] = 0.2 (units in millivolts), the parameters being such that $E\left[w\right]=\kappa \theta$, Var[*w*] = *κθ*^2^ (the three networks only differ in the value of the mean weight). (B) Mean ± standard deviation of the firing rate distribution at equilibrium as a function of the external firing rate *ν*_ext_. (C) Complete firing rate histogram in the three networks for three values of the external firing rate: 7.0 Hz (left), 7.5 Hz (middle), and 8.5 Hz (right). In all the plots, the empirical results come from integrating the full neuronal dynamics on networks with *N* = 1,000 neurons. The analytical results are obtained by numerically solving Equation 43 on ***θ*** = (*m*, *s*^2^). The neuronal parameters are: *K* = 25 (in-degree), *τ* = 20 ms, *V*_*θ*_ = 20 mV, *V*_*r*_ = 10 mV, *τ*_*r*_ = 2 ms, *K*_ext_ = 1,000, and *w*_ext_ = 0.14 mV.

To compare this result with the stationary rate distribution obtained from simulating the whole network, we took a network composed of *N* = 1,000 I neurons with fixed in-degree *K* = 25. The incoming neighbors were chosen randomly, resulting in normally distributed out-degrees (Figure 3A). The synaptic weights were taken from a gamma distribution. Figure 3 shows the comparison as we vary the weight expectation $E\left[w\right]$. The mean and standard deviation of the rate distribution (Figure 3B) and the rate distribution itself (Figure 3C) are well predicted by the theory, for different values of the external firing rate *ν*_ext_.

### Heterogeneous Network with Plastic Synaptic Weights (Model B)

We now consider a more complex scenario in which the network structure is determined not only by a heterogeneous connectivity defined by a joint distribution of in-/out-degrees but also by a plasticity mechanism that shapes individual synaptic weights. As stated in previous sections, the plasticity rule is such that, once the network reaches a stationary state, every synaptic weight *w*_*ij*_ is related to the pre- and postsynaptic firing rates *ν_j_, ν_i_* through

$$w_{ij}=g^{pre}\left(\nu_{j}\right)\;g^{\text{post}}\left(\nu_{i}\right)$$ (44)

for arbitrary functions *g*^pre^ and *g*^post^. This relationship is an approximation that becomes more accurate as the products *τ_p_ν_i_* and *τ_p_ν_j_* increase.

As in the previous cases, the stationary firing rate of a random neuron *i* is given by Equations 24 and 25. Again, we redefine the indices of the presynaptic neurons to neuron *i* to be 1, …, *K*_*i*_, with $K_{i}=K_{i}^{\text{in}}$ the in-degree of *i*, and we use Equation 44 to rewrite Equation 25 as

$$\begin{array}{lll} \mu_{i}=\tau \left(g^{\text{post}}\left(\nu_{i}\right)S_{\mu }^{i}+K_{\mathrm{ext}}w_{\mathrm{ext}}\nu_{\mathrm{ext}}\right)\\ \sigma_{i}^{2}=\tau \left(g^{\text{post}}{\left(\nu_{i}\right)}^{2}S_{\sigma }^{i}+K_{\mathrm{ext}}w_{\mathrm{ext}}^{2}\nu_{\mathrm{ext}}\right), & & \end{array}$$ (45)

where

$$\begin{array}{l} S_{\mu }^{i}≔\sum_{j=1}^{K_{i}}g^{pre}\left(\nu_{j}\right)\nu_{j}\\ S_{\sigma }^{i}≔\sum_{j=1}^{K_{i}}g^{pre}{\left(\nu_{j}\right)}^{2}\nu_{j}. \end{array}$$ (46)

The specific form of the weight-rate relationship defined by Equation 44 allows us to separate the pre- and postsynaptic components of each synaptic weight so that the sums $S_{\mu }^{i}$ and $S_{\sigma }^{i}$ do not depend on the postsynaptic rate *ν_i_*. As before, the vector

$$S_{i}≔\left(\begin{array}{l} S_{\mu }^{i}\\ S_{\sigma }^{i} \end{array}\right)=\sum_{j=1}^{K_{i}}\left(\begin{array}{l} g^{pre}\left(\nu_{j}\right)\nu_{j}\\ g^{pre}{\left(\nu_{j}\right)}^{2}\nu_{j} \end{array}\right)$$ (47)

can be assumed to be a sum over *K*_*i*_ i.i.d. random vectors $v_{1}^{i},\ldots ,v_{K_{i}}^{i}$,

$$v_{j}^{i}=\left(\begin{array}{l} g^{pre}\left(\nu_{j}\right)\nu_{j}\\ g^{pre}{\left(\nu_{j}\right)}^{2}\nu_{j} \end{array}\right),$$ (48)

and the distribution of $v_{j}^{i}$ is independent of the postsynaptic neuron *i* (and, thus, a *network* feature). We now define

$$m=\left(\begin{array}{l} m_{\mu }\\ m_{\sigma } \end{array}\right),\;\Sigma =\left(\begin{array}{ll} s_{\mu }^{2} & c_{\mu_{\sigma }}\\ c_{\mu_{\sigma }} & s_{\sigma }^{2} \end{array}\right)$$ (49)

as the mean vector and the covariance matrix of $v_{j}^{i}$:

$$\begin{array}{l} m_{\mu }≔E\left[\;g^{pre}\left(\nu_{j}\right)\nu_{j}\;|\;j\to i\;\right]\\ s_{\mu }^{2}≔Var\left[\;g^{pre}\left(\nu_{j}\right)\nu_{j}\;|\;j\to i\;\right]\\ m_{\sigma }≔E\left[\;g^{pre}{\left(\nu_{j}\right)}^{2}\nu_{j}\;|\;j\to i\;\right]\\ s_{\sigma }^{2}≔Var\left[\;g^{pre}{\left(\nu_{j}\right)}^{2}\nu_{j}\;|\;j\to i\;\right]\\ c_{\mu \sigma }≔Cov\left[\;g^{pre}\left(\nu_{j}\right)\nu_{j},g^{pre}{\left(\nu_{j}\right)}^{2}\nu_{j}\;|\;j\to i\;\right]. \end{array}$$ (50)

When *K*_*i*_ is known and large enough, the multidimensional version of the CLT ensures that the vector **S***_i_* will be approximately distributed as a bivariate normal vector with mean vector *K*_*i*_***m*** and covariance matrix *K*_*i*_Σ:

$$\begin{array}{lll} S_{i} & = & \left(\begin{array}{c} S_{\mu }^{i}\\ S_{\sigma }^{i} \end{array}\right)=K_{i}\left(\begin{array}{c} m_{\mu }\\ m_{\sigma } \end{array}\right)+\sqrt{K_{i}}\left(\begin{array}{c} Y_{i}\\ Z_{i} \end{array}\right), \end{array}$$ (51)

$$\begin{array}{lllllll} \left(\begin{array}{c} Y_{i}\\ Z_{i} \end{array}\right) & ∼ & N\left(0,\Sigma \right). & & & & \end{array}$$ (52)

Now, the set of mean-field parameters is $\theta ≔\left(m_{\mu },s_{\mu }^{2},m_{\sigma },s_{\sigma }^{2},c_{\mu_{\sigma }}\right)$. Once ***θ*** is known, the distribution of ***S****_i_* is determined through Equations 49, 51, and 52. The firing rate *ν_i_* of a neuron *i* is again determined by ***θ*** and by a triplet of identity variables associated to that neuron, ***X****_i_* = (*K*_*i*_, *Y*_*i*_, *Z*_*i*_) (whose distribution depends on the mean-field parameters). Due to the dependence of the synaptic weight *w*_*ij*_ on the postynaptic rate *ν_i_*, now, the way to compute *ν_i_* from ***θ*** and ***X****_i_* is no longer based on evaluating a function. Instead, one must solve a one-dimensional equation on *ν_i_*:

$$\nu_{i}=\phi \left(\mu \left(\nu_{i},\theta ,X_{i}\right),\sigma \left(\nu_{i},\theta ,X_{i}\right)\right),$$ (53a)

$$\begin{array}{lll} \mu \left(\nu_{i},\theta ,X_{i}\right)=\tau \left[g^{\text{post}}\left(\nu_{i}\right)\left(K_{i}\;m_{\mu }+\sqrt{K_{i}}Y_{i}\right)\right.\\ & & \left.+K_{\mathrm{ext}}w_{\mathrm{ext}}\nu_{\mathrm{ext}}\right]\\ \sigma^{2}\left(\nu_{i},\theta ,X_{i}\right)=\tau \left[g^{\text{post}}{\left(\nu_{i}\right)}^{2}\left(K_{i}\;m_{\sigma }+\sqrt{K_{i}}Z_{i}\right)\right.\\ & & \left.+K_{\mathrm{ext}}w_{\mathrm{ext}}^{2}\nu_{\mathrm{ext}}\right]. \end{array}$$ (53b)

We denote by Φ = Φ(***θ***, ***X****_i_*) a mapping that, given ***θ*** and ***X****_i_*, returns a solution to Equation 53 (a and b) on the unknown *ν_i_*.

As in the previous case, the identity variables *K*_*i*_, *Y*_*i*_, *Z*_*i*_ are random: *K*_*i*_ is distributed according to the in-degree distribution imposed in the network and (*Y*_*i*_, *Z*_*i*_) is a normal bivariate vector with zero mean and covariance matrix **Σ** = **Σ**(***θ***) (see Equation 52). The vector (*Y*_*i*_, *Z*_*i*_) is independent of *K*_*i*_, and the identity vectors of all the neurons, ***X***_1_, … , ***X****_N_*, are i.i.d.

The firing rate distribution can thus be computed once the mean-field parameter set $\theta =\left(m_{\mu },s_{\mu }^{2},m_{\sigma },s_{\sigma }^{2},c_{\mu_{\sigma }}\right)$ is known. By definition, the parameters in ***θ*** fulfill

$$\begin{array}{l} m_{\mu }=\int_{0}^{\infty }\int_{-\infty }^{\infty }\int_{-\infty }^{\infty }g^{pre}\left(\Phi \left(\theta ,x\right)\right)\Phi \left(\theta ,x\right)\rho_{X}^{\theta }\left(x\right)dx≕G_{m_{\mu }}\left(\theta \right)\\ s_{\mu }^{2}=\int_{0}^{\infty }\int_{-\infty }^{\infty }\int_{-\infty }^{\infty }{\left[g^{pre}\left(\Phi \left(\theta ,x\right)\right)\Phi \left(\theta ,x\right)-m_{\mu }\right]}^{2}\rho_{X}^{\theta }\left(x\right)dx≕G_{s_{\mu }^{2}}\left(\theta \right)\\ m_{\sigma }=\int_{0}^{\infty }\int_{-\infty }^{\infty }\int_{-\infty }^{\infty }g^{pre}{\left(\Phi \left(\theta ,x\right)\right)}^{2}\Phi \left(\theta ,x\right)\rho_{X}^{\theta }\left(x\right)dx≕G_{m_{\sigma }}\left(\theta \right)\\ s_{\sigma }^{2}=\int_{0}^{\infty }\int_{-\infty }^{\infty }\int_{-\infty }^{\infty }{\left[g^{pre}{\left(\Phi \left(\theta ,x\right)\right)}^{2}\;\Phi \left(\theta ,x\right)-m_{\sigma }\right]}^{2}\rho_{X}^{\theta }\left(x\right)dx≕G_{s_{\sigma }^{2}}\left(\theta \right)\\ c_{\mu \sigma }=\int_{0}^{\infty }\int_{-\infty }^{\infty }\int_{-\infty }^{\infty }\left[g^{pre}\left(\Phi \left(\theta ,x\right)\right)\Phi \left(\theta ,x\right)-m_{\mu }\right]\left[g^{pre}{\left(\Phi \left(\theta ,x\right)\right)}^{2}\;\Phi \left(\theta ,x\right)-m_{\sigma }\right]\rho_{X}^{\theta }\left(x\right)\;dx≕G_{c_{\mu \sigma }}\left(\theta \right), \end{array}$$ (54)

where ***x*** = (*k*, *y*, *z*) and $\rho_{X}^{\theta }\left(x\right)$ is the joint p.d.f. of the triplet ***X***_*i*_ = (*K*_*i*_, *Y*_*i*_, *Z*_*i*_) for a presynaptic neuron:

$$\rho_{X}^{\theta }\left(x\right)=\rho_{K}^{pre}\left(k\right)\;\rho_{Y,Z}^{\theta }\left(y,z\right),$$ (55)

with $\rho_{K}^{pre}$ being the p.d.f. of the in-degree of a presynaptic neuron (see Supporting Information Section S3.3) and $\rho_{Y,Z}^{\theta }$ being the p.d.f. of a normal bivariate vector with mean **0** and covariance matrix Σ(***θ***).

The mean-field parameter set ***θ*** is thus found by solving the system of five unknowns and five equations

$$\theta =G\left(\theta \right),$$ (56)

where $G\left(\theta \right)≔\left(G_{m_{\mu }}G_{s_{\mu }^{2}},G_{m_{\sigma }},G_{s_{\sigma }^{2}},G_{c_{\mu \sigma }}\right)\left(\theta \right)$ and the component functions are defined in Equation 54.

The firing rate distribution can be reconstructed analogously as we did for Model A. In this scenario, we are also interested in anticipating the distribution of synaptic weights. Once the system Equation 56 is solved and we know the value of ***θ***, the synaptic weight of a randomly chosen connection is computed as follows. Calling *i* and *j* the post- and presynaptic neurons involved in the connection, respectively, with identity variables ***X****_i_* = (*K*_*i*_, *Y*_*i*_, *Z*_*i*_) and ***X****_j_* = (*K*_*j*_, *Y*_*j*_, *Z*_*j*_), the firing rates of *i* and *j* are

$$\nu_{i}=\Phi \left(\theta ,X_{i}\right),\;\nu_{j}=\Phi \left(\theta ,X_{j}\right).$$ (57)

The synaptic weight of the connection *i* ← *j* is, then, given by Equation 44. Note, however, that the in-degrees *K*_*i*_ and *K*_*j*_ do not necessarily follow the in-degree distribution imposed in the network: The fact that a connection exists from *j* to *i* always biases the in-degree distribution of *i* and can bias the in-degree distribution of *j* (if individual in-/out-degrees are correlated). As detailed in Supporting Information Section S3, these distributions are specified by

$$\begin{array}{lll} P\left(K_{i}^{\text{in}}=k\;|\;i\leftarrow j\right) & = & \frac{k}{\langle K\rangle }P\left(K_{i}^{\text{in}}=k\right)\\ P\left(K_{j}^{\text{in}}=m\;|\;i\leftarrow j\right) & = & \frac{\langle K^{\text{out}}\;|\;K^{\text{in}}=m\rangle }{\langle K\rangle }\;P\left(K_{j}^{\text{in}}=m\right), \end{array}$$ (58)

where 〈*K*〉 and 〈*K*^out^ | *K*^in^ = *m*〉 are, respectively, the expected (in- and out-) degree and the expected out-degree of a neuron conditioned to its in-degree being *m*. Equation 58 shows that the in-degree distribution for a postsynaptic neuron is always biased with respect to the network in-degree distribution. The in-degree distribution for a presynaptic neuron is only biased when individual in- and out-degrees in the network are correlated. To numerically reconstruct the weight distribution, we can create a large sample of pre- and postsynaptic triplets ***X****_j_* and ***X****_i_*, taking into account the pre- and postsynaptic degree distributions given in Equation 58 and then use it to create a sample of synaptic weights through Equations 57 and 44.

This formalism can be extended to networks composed of E and I neurons as we detail in Supporting Information Sections S4 and S5.

We verified that the described equations can predict the weight and firing rate distributions in the stationary state. For this, we first simulated the microscopic dynamics of a network composed of *N*_*E*_ excitatory and *N*_*I*_ inhibitory neurons with *N*_*E*_ = *N*_*I*_ = 1,000 in which all the synaptic weights were plastic. The plasticity rule for E synapses was inspired by Oja’s rule (Oja, 1982), see Equations 7, 10, and 12. The I rule was taken to be analogous but with the opposite sign to simplify the resulting mean-field equations.

**Figure 4.**
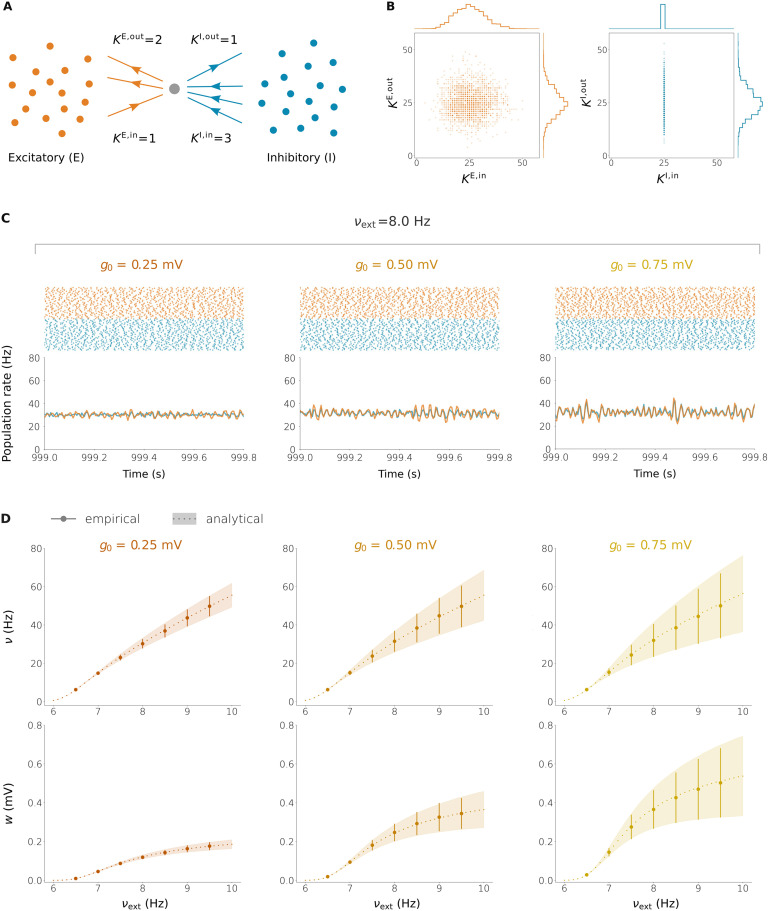
Firing rate and synaptic weight statistics in E-I networks with plastic synaptic weights. (A) Each neuron in the network has four characteristic degrees: the in-/out-degrees coming from/going to the E and I populations (*K*^*E*,in^, *K*^*E*,out^, *K*^*I*,in^, *K*^*I*,out^). (B) E and I in-/out-degree histogram (statistically identical in all the networks), with normally distributed E in-degrees, E out-degrees, and I out-degrees and a fixed I in-degree. The four degrees associated to each neuron are independent random variables. (C) Spike times (top) and instantaneous population firing rates (bottom) when *ν*_ext_ = 8.0 Hz for three choices of the plasticity parameter *g*_0_ (see Equations 10, 12). (D) Mean ± standard deviation of the E/I firing rate (top) and E synaptic weight (bottom) distributions at equilibrium as a function of the external firing rate *ν*_ext_ for the same three networks of (C). In all the plots, the empirical results come from integrating the full neuronal dynamics on networks with *N*_*E*_ = *N*_*I*_ = 1,000 neurons. The analytical results are obtained by numerically solving Equation 56 on $\theta =\left(m_{\mu },s_{\mu }^{2},m_{\sigma },s_{\sigma }^{2},c_{\mu \sigma }\right)$. The E in-/out-degrees are $K_{i}^{E,\text{in}},K_{i}^{E,\text{out}}∼N\left(\mu_{K},\sigma_{K}\right)$ with *μ*_*K*_ = 25, *σ*_*K*_ = 7. The I in-degree is the same for all the neurons, $K_{i}^{I,\text{in}}=25$. The neuronal parameters are *τ* = 20 ms, *V*_*θ*_ = 20 mV, *V*_*r*_ = 10 mV, *τ*_*r*_ = 2 ms, *K*_ext_ = 1,000, and *w*_ext_ = 0.14 mV. The remaining plasticity parameters are *ε* = 0.001 ms^−2^ and *τ*_*p*_ = 50 s.

**Figure 5.**
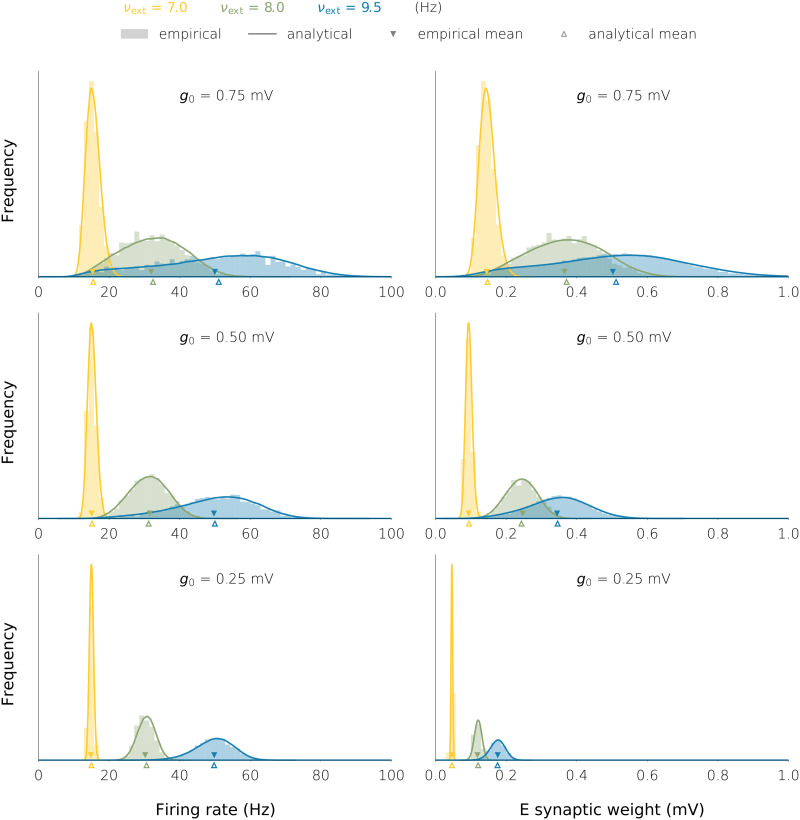
E/I firing rate and E synaptic weight histograms in the three networks with plastic synaptic weights of Figure 4. The histograms are shown for the three choices of the external firing rate.

In our example network, degrees from/to the E population were normally distributed and independent, whereas the in-degrees from the I population were fixed (the I incoming neighbors were chosen randomly, resulting in normally distributed I out-degrees); see Figure 4A, B. The reason to include I neurons to the network of E neurons is that the network should be approximately balanced for it to reach a stationary state with irregular (and, hence, close to Poisson) firing and low firing rates. The raster plots in Figure 4C show this irregular firing. Figure 4D shows the mean and standard deviation of the rate and weight distributions as the external firing rate is increased, for three choices of the plasticity parameter *g*_0_ (see Equation 12). A sample of the corresponding distributions is given in Figure 5, showing a very good agreement between theory and simulations.

**Figure 6.**
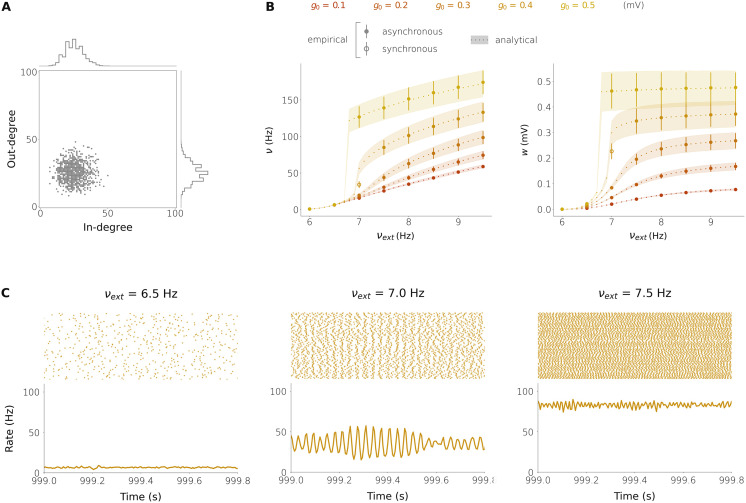
Firing rate and synaptic weight statistics in five excitatory networks with plastic synaptic weights. (A) In-/out-degree histogram (the same in all the networks), with normally distributed in-degree and out-degree and no correlation between individual degrees. (B) Mean ± standard deviation of the firing rate (left) and synaptic weight (right) distributions at equilibrium as a function of the external firing rate *ν*_ext_. The five networks only differ in the parameter *g*_0_ of the plasticity rule (see Equations 10, 12). (C) Spike times (top) and instantaneous population firing rate (bottom) for the network with *g*_0_ = 0.4 mV for the three choices of *ν*_ext_. In all the plots, the empirical results come from integrating the full neuronal dynamics on networks with *N* = 1,000 neurons. The analytical results are obtained by numerically solving Equation 56 on $\theta =\left(m_{\mu },s_{\mu }^{2},m_{\sigma },s_{\sigma }^{2},c_{\mu \sigma }\right)$. The in-/out-degrees are $K_{i}^{\text{in}},K_{i}^{\text{out}}∼N\left(\mu_{K},\sigma_{K}\right)$ with *μ*_*K*_ = 25, *σ*_*K*_ = 7. The neuronal parameters are *τ* = 20 ms, *V*_*θ*_ = 20 mV, *V*_*r*_ = 10 mV, *τ*_*r*_ = 2 ms, *K*_ext_ = 1,000, and *w*_ext_ = 0.14 mV. The remaining plasticity parameters are *ε* = 0.001 ms^−2^ and *τ*_*p*_ = 50 s.

**Figure 7.**
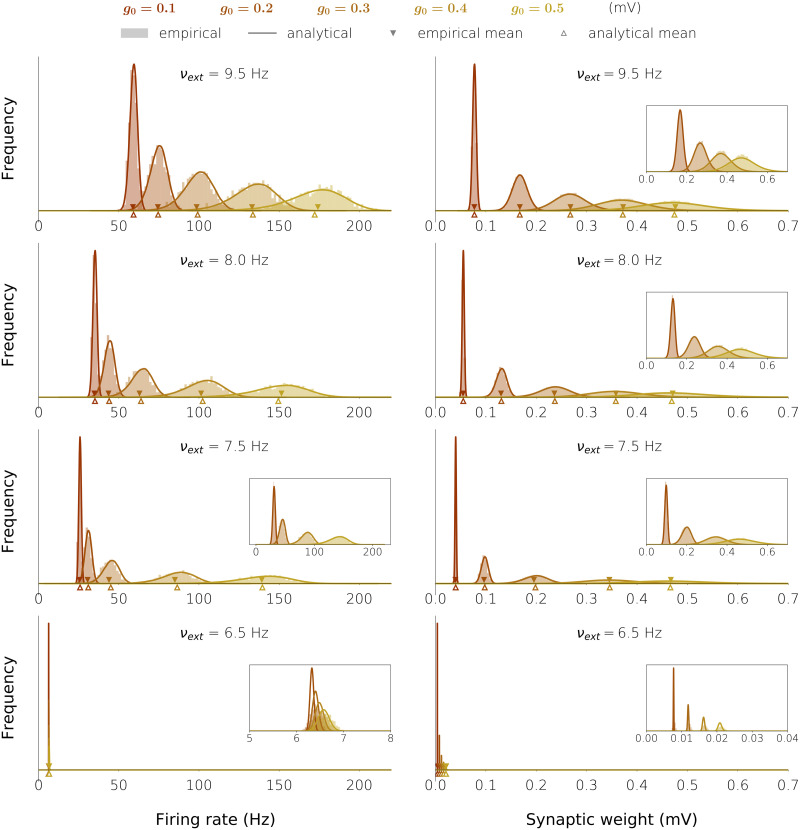
Firing rate and synaptic weight histograms in the five excitatory networks with plastic synaptic weights. The networks are those of Figure 6, and the histograms are shown for the four values of the external firing rate: 6.5, 7.5, 8.0, and 9.5 Hz.

We also investigated to what extent this agreement can be extended to plastic networks composed solely of E neurons. In a network of this kind, if the external firing rate is large enough, the hypothesis of Poissonian firing cannot be guaranteed, and this can make the network be outside of the parameter range in which the analytical solution is correct. Surprisingly, we found that for many choices of the external rate, the analytical prediction matches the simulations quite well; see Figures 6B, C and 7. However, there seems to be a restricted range in the external rate for which the network activity has some degree of synchrony and regular firing, and in this case, the analytical prediction does not match the empirical results. This is the case of the network with *g*_0_ = 0.4 mV and *ν*_ext_ = 7 Hz in Figure 6B,C. This range coincides with the range in which the network activity shifts from a low firing to a high firing state (Figure 6B).

## DISCUSSION

We have derived of a set of mean-field equations that bridge the gap between a microscopical and a macroscopical description of the neuronal activity in a heterogeneous network of LIF spiking neurons at equilibrium. Whereas the microscopical description is given in terms of membrane voltages and spike times, in the macroscopical description, the neuronal activity is represented by the neurons’ firing rates (i.e., average number of spikes emitted per unit time). Although this kind of mean-field formalism has been widely used before, the main contribution of the present work has been to generalize it so that it is applicable to networks in which two sources of structural heterogeneity may take place at the same time: a heterogeneity in terms of in- and out-degrees and a heterogeneity in terms of synaptic weights, including weights that have been shaped by an activity-dependent plasticity mechanism.

In the nonplastic scenario, the synaptic weights were assumed to be independent variables from a common probability distribution. In the model with plasticity, every neuron had associated a spike trace (which could represent the concentration of a chemical that increases every time the neuron emits a spike and which is degraded over time; see Pfister & Gerstner, 2006 for possible interpretations), and the instantaneous variation of every synaptic weight was a function of the pre- and postsynaptic traces. We assumed that the network was on a regime in which, at equilibrium, the traces’ fluctuations around their means are small so that they can be used to approximate the neurons’ firing rates. This is the key step to include the plasticity mechanism into the mean-field equations, because at equilibrium, every synaptic weight can be considered to be a known function of the pre- and postsynaptic firing rates.

Given a (postsynaptic) LIF neuron, its firing rate at equilibrium is a well-defined function of its presynaptic neighbors’ rates and the corresponding synaptic weights. More precisely, it is a function of two important quantities: the sum (over the presynaptic neighbors) of the presynaptic rates times the weights, *S_μ_*, and the sum of the presynaptic rates times the squares of the weights, *S_σ_*. The firing rates (and the synaptic weights in the plastic model) are, however, not known a priori: The purpose is precisely to compute them analytically. For this, another key step is necessary: Under reasonable hypotheses, the aforementioned sums can be assumed to be sums over identically distributed random variables, which allows us to apply the CTL to deduce that they are jointly normally distributed. This step reduces the complexity of the problem from computing the *whole* rate/weight distribution to computing just *a few* statistical parameters that characterize the normal vector (*S_μ_*, *S_σ_*). These parameters are computed by invoking their definitions as statistics related to the firing rate distribution, which gives a set of equations on the parameters themselves that can be solved numerically. The results seem to match well with direct simulations of the microscopic dynamics on networks composed of both I and I-E LIF neurons.

This work is, to our knowledge, the first to simultaneously tackle the problem of extending previous mean-field formalisms to networks in which there is a heterogeneity of both degrees and synaptic weights, including weights that are plastic. It thus offers a step forward in the tremendous effort for understanding and predicting the collective behavior of networks of LIF neurons in these scenarios.

Our work has, however, several limitations that should be pointed out. We considered networks composed of LIF neurons because the LIF model is simpler and more amenable to analytical treatment than more realistic models. However, the LIF model is unable to reproduce some of the electrophysiological properties found in real neurons. The effective threshold for firing in real neurons, for example, seems to be not fixed but to depend on the stimulation protocol (Mensi, Hagens, Gerstner, & Pozzorini, 2016), and this can be reproduced by nonlinear IF models like the quadratic (Latham, Richmond, Nelson, & Nirenberg, 2000) or the exponential (Fourcaud-Trocmé, Hansel, van Vreeswijk, & Brunel, 2003) models. Another example is the spike-triggered adaptation, a process by which the spike frequency decreases upon sustained firing. Models of IF neurons including spike-triggered and subthreshold adaptation by means of an additional dynamic variable have been shown to be notably more realistic (Brette & Gerstner, 2005; Hertäg, Hass, Golovko, & Durstewitz, 2012; Izhikevich, 2004; Naud, Marcille, Clopath, & Gerstner, 2008) while still being simple compared with detailed biophysical models like the Hodgkin-Huxley model (Hodgkin & Huxley, 1952). Despite these, nonlinear and adaptive models have been successively studied under the lens of mean-field techniques (Brunel, Hakim, & Richardson, 2003; Brunel & Latham, 2003; Fourcaud-Trocmé et al., 2003; Hertäg, Durstewitz, & Brunel, 2014; Montbrió, Pazó, & Roxin, 2015); to what extent the analysis performed here could be extended to them, too, remains an open question.

Another important limitation of our work concerns the plasticity rule. Since the neuronal activity in the microscopic model is given by the spike train, any plasticity rule implemented on such a network should be a spike-dependent rule or, at a slightly more coarse-grained scale, a burst-dependent rule (Payeur, Guerguiev, Zenke, Richards, & Naud, 2021). The mean-field description is, however, given in terms of firing rates, and this is why going from one description to the other requires rewriting the plasticity rule at equilibrium in terms of firing rates. A natural way to do so is by considering spike traces: stochastic variables reflecting the spiking activity whose statistics, as we showed, can be directly linked to the underlying firing rates. However, in our mean-field formalism, it is assumed that the synaptic weight at equilibrium is fixed once the firing rate is known, and this does not allow for the introduction of weight fluctuations caused by the traces’ fluctuations. The parameter regime in which the spike trace is a reliable estimator of the firing rate (i.e., the regime in which the trace’s fluctuations are small compared with their average) is precisely the regime at which the product of the firing rate and the trace degradation constant *τ_p_* is large. This limits the applicability range of our mean-field formulation and, particularly, makes it not applicable when the plasticity rule in place is a function of *both* the traces and the spike trains themselves or a function of several traces per neuron, with different characteristic degradation constants, as in pair-based and triplet STDP rules (Morrison et al., 2008; Pfister & Gerstner, 2006). One further step would be to study if not only the trace averages but also their fluctuations could be taken into account in the mean-field formalism. In this case, every synaptic weight at a given time would be a stochastic variable, whose statistics at equilibrium should be introduced in the mean-field formulation.

A central hypothesis in our mean-field equations is that the network’s structure is such that the in-degrees of two connected neurons are independent variables. This implies that the distribution of in-degrees among the presynaptic neurons to a given postsynaptic neuron is the same for all postsynaptic neurons: It is a *network* property. This ingredient is central to reduce the space of unknowns down to a set of a few parameters, because we use the fact that the statistics of every input to a neuron are independent of the identity of that neuron. It would be interesting to study how our theory should be modified so as to include assortative or dissortative networks, as was done for assortative networks with homogeneous synaptic weights in Schmeltzer, Kihara, Sokolov, and Rüdiger (2015).

Finally, we did not analyze the stability of the stationary distribution of firing rates predicted by the theory, or whether there is more than one stationary distribution depending on the model’s parameters. We leave such questions for the future.

## ACKNOWLEDGMENTS

We acknowledge Calcul Québec and the Digital Research Alliance of Canada for their technical support and computing infrastructures.

## SUPPORTING INFORMATION

Supporting information for this article is available at https://doi.org/10.1162/netn_a_00442.

## AUTHOR CONTRIBUTIONS

Marina Vegué: Conceptualization; Formal analysis; Investigation; Methodology; Software; Writing – original draft; Writing – review & editing. Antoine Allard: Funding acquisition; Project administration; Writing – review & editing. Patrick Desrosiers: Conceptualization; Funding acquisition; Project administration; Writing – review & editing.

## FUNDING INFORMATION

Antoine Allard, Natural Sciences and Engineering Research Council of Canada (https://dx.doi.org/10.13039/501100000038). Patrick Desrosiers, Sentinelle Nord, Université Laval (https://dx.doi.org/10.13039/100020862). Marina Vegué, Ministerio de Universidades (https://dx.doi.org/10.13039/501100023561). Patrick Desrosiers, Natural Sciences and Engineering Research Council of Canada (https://dx.doi.org/10.13039/501100000038). Antoine Allard, Sentinelle Nord, Université Laval (https://dx.doi.org/10.13039/100020862).

## Supplementary Material



## TECHNICAL TERMS

Integrate-and-fire model: Model for the activity of a neuron that is formulated through (a) a differential equation describing the temporal evolution of the membrane potential V and (b) a mechanism for spike generation that depends on V.
Spike train: Collection of spike times for a given neuron.
Mean-field theory: Mathematical theory for studying stochastic interacting systems composed of statistically identical units that allows to describe the evolution of the ensemble’s statistics using a simpler model.
Stationary state: State that is stable in time.
Firing rate: The average number of spikes emitted by a neuron per unit time.
Joint in-/out-degree distribution: Distribution of the degree pair (in-degree, out-degree) associated to a neuron in a random network model.
Structural plasticity: Modification of the connectivity structure (“who connects to whom”) in an interacting network.
Synaptic plasticity: Modification of the synaptic weights in a neuronal network.
Spike-timing-dependent plasticity (STDP): Model of synaptic plasticity in which the evolution of every synaptic weight depends on the specific timing of pre- and postsynaptic spikes emitted by the two neurons involved in the connection.
