# Supplementary material for: Firing rate distributions in plastic networks of spiking neurons
Source: Netw Neurosci. 2025 Mar 20;9(1):447–74. doi: 10.1162/netn_a_00442 (PMC11949577; doi:10.1162/netn_a_00442)
Supplement: Supplementary file 1 [file netn-9-1-447-s001.pdf]

Firing rate distributions in plastic networks of spiking neurons

Marina Vegué, Antoine Allard, and Patrick Desrosiers

— Supplementary Materials —

1 MATHEMATICAL ANALYSIS OF THE SPIKE TRACE

We consider the spike trace R of a neuron to evolve in time according to

$$\frac{\mathrm{d}}{\mathrm{d}t}R(t) = -\frac{1}{\tau_p}R(t) + \sum_k \delta(t - t^k), \tag{S1}$$

where $t^1, \cdots, t^k$ are the times at which the neuron has spiked in the past. Eq. (S1) essentially states that the variable R decays exponentially to 0 with a characteristic time scale τ_p and makes jumps of magnitude 1 every time there is a spike.

1.1 Probability density function of R

If the neuron fires as a Poisson process of rate ν, Eq. (S1) is a stochastic differential equation and $R(t)$ is a random variable. Moreover, R has the Markov property. Denoting by $\rho(r,t)$ the probability density function of $R(t)$ for a fixed initial condition, ρ obeys the so-called forward Smolukowski equation [1]:

$$\frac{\partial}{\partial t}\rho(r,t) = \sum_{n=1}^{\infty} \frac{(-1)^n}{n!} \frac{\partial^n}{\partial r^n} \left[A_n(r)\rho(r,t)\right], \tag{S2}$$

where

$$A_n(r) := \lim_{\Delta t \to 0^+} \frac{1}{\Delta t} \int_{-\infty}^{\infty} (r' - r)^n \rho(r', t + \Delta t \,|\, r, t)\, \mathrm{d}r' \tag{S3}$$

is the n-th *infinitesimal moment* of R and $\rho(r', t+\Delta t \,|\, r, t)$ denotes its transition p.d.f. from state r at time t to state r' at time $t + \Delta t$ [1]. Following [2] (chapter 15), the infinitesimal moments can be computed as follows.

Let Δt be small enough so that the probability that the neuron spikes more than once within a time window of length Δt is negligible. Then, in this time window, either:

1. one spike is emitted, with probability $\Delta t\, \nu$;
2. no spikes are emitted, with probability $1 - \Delta t\, \nu$.

In these scenarios, the value $R(t + \Delta t) = r_{1,0}$ can be explicitly computed from $R(t) = r$:

1. $r_1 = (re^{-\Delta t'/\tau_p} + 1)\, e^{-(\Delta t - \Delta t')/\tau_p}$, where $\Delta t' \leq \Delta t$ is the time lapse until the spike was emitted;
2. $r_0 = re^{-\Delta t/\tau_p}$.

Thus, if $\Delta t'$ were known, we would have

$$\rho(r', t + \Delta t \,|\, r, t) = (1 - \Delta t\, \nu)\, \delta(r' - r_0) + \Delta t\, \nu\, \delta(r' - r_1), \tag{S4}$$

δ being the Dirac delta distribution. Eq. (S4) allows us to compute the limit in Eq. (S3) to get

$$A_n(r) = \begin{cases} -\dfrac{r}{\tau_p} + \nu & \text{if } n = 1, \\ \nu & \text{if } n \geq 2. \end{cases} \tag{S5}$$

Eq. (S2) is then equivalent to

$$\begin{aligned} \frac{\partial}{\partial t}\rho(r,t) &= \frac{\partial}{\partial r}\left[\frac{r}{\tau_p}\rho(r,t)\right] + \nu \sum_{n=1}^{\infty} \frac{(-1)^n}{n!}\frac{\partial^n}{\partial r^n}\rho(r,t) \\ &= \frac{\partial}{\partial r}\left[\frac{r}{\tau_p}\rho(r,t)\right] - \nu\rho(r,t) + \nu \sum_{n=0}^{\infty} \frac{(-1)^n}{n!}\frac{\partial^n}{\partial r^n}\rho(r,t). \end{aligned} \tag{S6}$$

The infinite sum at the end is the Taylor expansion of $\rho(r-1,t)$ around r. Assuming that this expansion converges, we can rewrite the previous equation as

$$\frac{\partial}{\partial t}\rho(r,t) = \left(\frac{1}{\tau_p} - \nu\right)\rho(r,t) + \frac{r}{\tau_p}\frac{\partial}{\partial r}\rho(r,t) + \nu\rho(r-1,t). \tag{S7}$$

This can in turn be rewritten as

$$\tau_p \frac{\partial}{\partial t}\rho(r,t) = (1-\alpha)\,\rho(r,t) + r\frac{\partial}{\partial r}\rho(r,t) + \alpha\rho(r-1,t) \tag{S8}$$

with $\alpha := \tau_p \nu$. In particular, the stationary distribution of r, $\rho(r)$, fulfills

$$r\rho'(r) = (\alpha - 1)\,\rho(r) - \alpha\rho(r-1). \tag{S9}$$

1.2 Recursive ODEs for the moments of R

We denote by $\langle R\rangle(t)$ the expectation of $R(t)$ and by $\langle R_n\rangle(t)$ the centered moment of order $n \geq 0$ of $R(t)$:

$$\begin{aligned} \langle R\rangle(t) &:= \int_{-\infty}^{\infty} r\rho(r,t)\,\mathrm{d}r \\ \langle R_n\rangle(t) &:= \int_{-\infty}^{\infty} \left[r - \langle R\rangle(t)\right]^n \rho(r,t)\,\mathrm{d}r \quad \text{for } n \geq 0. \end{aligned} \tag{S10}$$

Notice that $\langle R_0\rangle(t) = 1$ and $\langle R_1\rangle(t) = 0$ for all t.

Now we derive a recursive system of ordinary differential equations (ODEs) for the moments of R from the temporal evolution of R's density function [Eq. (S8)]. We assume the following property for ρ: for any t, the tails of the density $\rho(r,t)$ go to zero faster than any power of $1/r$, that is,

$$\lim_{r\to\pm\infty} r^k \rho(r,t) = 0 \quad \text{for any } k \geq 0. \tag{S11}$$

Also, we use the fact that if $f = f(r,t)$ is a differentiable function in r and ξ is an arbitrary constant, then, for any $k \neq 0$,

$$(r-\xi)^k \frac{\partial}{\partial r} f(r,t) = -k\,(r-\xi)^{k-1} f(r,t) + \frac{\partial}{\partial r}\left[(r-\xi)^k f(r,t)\right]. \tag{S12}$$

From this we deduce the following: if $f = f(r,t)$ is an arbitrary differentiable function in r and ξ, a, b are arbitrary constants, then, for any $k \neq 0$,

$$\int_a^b (r-\xi)^k \frac{\partial}{\partial r} f(r,t)\;\mathrm{d}r = -k\int_a^b (r-\xi)^{k-1} f(r,t)\;\mathrm{d}r + \left[(r-\xi)^k f(r,t)\right]_{r=a}^{r=b}. \tag{S13}$$

Property (S13) and assumption (S11) jointly imply that, for any $k > 0$ and any constant ξ,

$$\int_{-\infty}^{\infty} (r-\xi)^k \frac{\partial}{\partial r}\rho(r,t)\;\mathrm{d}r = -k\,\langle (R(t)-\xi)^{k-1}\rangle, \tag{S14}$$

where $\langle (R(t)-\xi)^{k-1}\rangle$ is the expectation of $(R(t)-\xi)^{k-1}$.

We start with the expectation of $R(t)$. Multiplying both sides of Eq. (S8) by r and integrating we have

$$\tau_p \int_{-\infty}^{\infty} r\frac{\partial}{\partial t}\rho(r,t)\;\mathrm{d}r = (1-\alpha)\int_{-\infty}^{\infty} r\rho(r,t)\;\mathrm{d}r + \int_{-\infty}^{\infty} r^2\frac{\partial}{\partial r}\rho(r,t)\;\mathrm{d}r + \alpha\int_{-\infty}^{\infty} r\rho(r-1,t)\;\mathrm{d}r. \tag{S15}$$

Using property (S14) and making a change of variables in the last integral we can rewrite this as

$$\begin{aligned} \tau_p\langle \dot{R}\rangle &= (1-\alpha)\langle R\rangle - 2\langle R\rangle + \alpha\int_{-\infty}^{\infty}(r+1)\rho(r,t)\;\mathrm{d}r \\ &= (1-\alpha)\langle R\rangle - 2\langle R\rangle + \alpha\left(\langle R\rangle + 1\right) \\ &= \alpha - \langle R\rangle. \end{aligned} \tag{S16}$$

We move to the centered moment $\langle R_n\rangle(t)$, $n \geq 0$. By construction, for all t, $\langle R_0\rangle(t) = 1$ and $\langle R_1\rangle(t) = 0$. For $n \geq 2$,

$$\begin{aligned} \tau_p\langle \dot{R_n}\rangle &= \tau_p\frac{\mathrm{d}}{\mathrm{d}t}\int_{-\infty}^{\infty}(r-\langle R\rangle)^n\rho(r,t)\;\mathrm{d}r \\ &= -n\tau_p\langle\dot{R}\rangle\int_{-\infty}^{\infty}(r-\langle R\rangle)^{n-1}\rho(r,t)\;\mathrm{d}r + \tau_p\int_{-\infty}^{\infty}(r-\langle R\rangle)^n\frac{\partial}{\partial t}\rho(r,t)\;\mathrm{d}r \\ &= -n\tau_p\langle\dot{R}\rangle\langle R_{n-1}\rangle + \tau_p\int_{-\infty}^{\infty}(r-\langle R\rangle)^n\frac{\partial}{\partial t}\rho(r,t)\;\mathrm{d}r. \end{aligned} \tag{S17}$$

Using Eq. (S8), the last integral is

$$\begin{aligned} \tau_p\int_{-\infty}^{\infty}(r-\langle R\rangle)^n\frac{\partial}{\partial t}\rho(r,t)\;\mathrm{d}r &= (1-\alpha)\int_{-\infty}^{\infty}(r-\langle R\rangle)^n\rho(r,t)\;\mathrm{d}r + \int_{-\infty}^{\infty}(r-\langle R\rangle)^n r\frac{\partial}{\partial r}\rho(r,t)\;\mathrm{d}r \\ &\quad +\alpha\int_{-\infty}^{\infty}(r-\langle R\rangle)^n\rho(r-1,t)\;\mathrm{d}r \\ &= (1-\alpha)\langle R_n\rangle + \int_{-\infty}^{\infty}(r-\langle R\rangle)^n r\frac{\partial}{\partial r}\rho(r,t)\;\mathrm{d}r \\ &\quad +\alpha\int_{-\infty}^{\infty}(r-\langle R\rangle)^n\rho(r-1,t)\;\mathrm{d}r. \end{aligned} \tag{S18}$$

We compute the two last integrals separately. Using property (S14), the first one is

$$\begin{aligned}\int_{-\infty}^{\infty}(r-\langle R\rangle)^n\, r\frac{\partial}{\partial r}\rho(r,t)\ \mathrm{d}r &= \int_{-\infty}^{\infty}(r-\langle R\rangle)^n\,(r-\langle R\rangle+\langle R\rangle)\,\frac{\partial}{\partial r}\rho(r,t)\ \mathrm{d}r\\ &= \int_{-\infty}^{\infty}(r-\langle R\rangle)^{n+1}\,\frac{\partial}{\partial r}\rho(r,t)\ \mathrm{d}r+\langle R\rangle\int_{-\infty}^{\infty}(r-\langle R\rangle)^n\,\frac{\partial}{\partial r}\rho(r,t)\ \mathrm{d}r\\ &= -(n+1)\langle R_n\rangle-n\langle R\rangle\langle R_{n-1}\rangle.\end{aligned}\tag{S19}$$

The second integral is

$$\begin{aligned}\int_{-\infty}^{\infty}(r-\langle R\rangle)^n\,\rho(r-1,t)\ \mathrm{d}r &= \int_{-\infty}^{\infty}(r-\langle R\rangle+1)^n\,\rho(r,t)\ \mathrm{d}r\\ &= \int_{-\infty}^{\infty}\sum_{k=0}^{n}\binom{n}{k}(r-\langle R\rangle)^k\,\rho(r,t)\ \mathrm{d}r\\ &= \sum_{k=0}^{n}\binom{n}{k}\langle R_k\rangle.\end{aligned}\tag{S20}$$

We obtain

$$\begin{aligned}\tau_p\langle\dot{R_n}\rangle &= -n\tau_p\langle\dot{R}\rangle\langle R_{n-1}\rangle+(1-\alpha)\langle R_n\rangle\\ &\quad -(n+1)\langle R_n\rangle-n\langle R\rangle\langle R_{n-1}\rangle+\alpha\sum_{k=0}^{n}\binom{n}{k}\langle R_k\rangle\\ &= -n\tau_p\frac{\mathrm{d}}{\mathrm{d}t}\langle r(t)\rangle\langle r_{n-1}(t)\rangle-(\alpha+n)\langle r_n(t)\rangle-n\langle r(t)\rangle\langle r_{n-1}(t)\rangle+\alpha\sum_{k=0}^{n}\binom{n}{k}\langle r_k(t)\rangle\\ &= -n\tau_p\langle\dot{R}\rangle\langle R_{n-1}\rangle-n\langle R_n\rangle-n\langle R\rangle\langle R_{n-1}\rangle+\alpha\sum_{k=0}^{n-1}\binom{n}{k}\langle R_k\rangle.\end{aligned}\tag{S21}$$

Finally, we use Eq. (S16) to obtain

$$\begin{aligned}\tau_p\langle\dot{R_n}\rangle &= -n\left(\alpha-\langle R\rangle\right)\langle R_{n-1}\rangle-n\langle R_n\rangle-n\langle R\rangle\langle R_{n-1}\rangle+\alpha\sum_{k=0}^{n-1}\binom{n}{k}\langle R_k\rangle\\ &= -n\alpha\langle R_{n-1}\rangle-n\langle R_n\rangle+\alpha\sum_{k=0}^{n-1}\binom{n}{k}\langle R_k\rangle\\ &= -n\langle R_n\rangle+\alpha\sum_{k=0}^{n-2}\binom{n}{k}\langle R_k\rangle.\end{aligned}\tag{S22}$$

Since $\langle R_0\rangle(t)\equiv 1$ and $\langle R_1\rangle(t)\equiv 0$, we can rewrite this as follows:

$$\tau_p\langle\dot{R_n}\rangle=\alpha-n\langle R_n\rangle+\alpha\sum_{k=2}^{n-2}\binom{n}{k}\langle R_k\rangle\qquad\text{for } n\geq 2.\tag{S23}$$

To wrap up, at time t, the centered moments of $R(t)$ evolve in time according to

$$\begin{aligned}\tau_p\langle\dot{R}\rangle &= \alpha-\langle R\rangle\\ \tau_p\langle\dot{R_n}\rangle &= \alpha-n\langle R_n\rangle+\alpha\sum_{k=2}^{n-2}\binom{n}{k}\langle R_k\rangle\qquad\text{for } n\geq 2.\end{aligned}\tag{S24}$$

1.3 Asymptotic behavior of the centered moments

Let us consider Eq. (S24) up to a fixed order $m\geq 2$. This is a system of m ordinary differential equations for the expectation and the centered moments of R up to order m. The system is affine: it has the form

$$\dot{\boldsymbol{x}}=\boldsymbol{\alpha}+\boldsymbol{M}\boldsymbol{x},\tag{S25}$$

where $\boldsymbol{x}(t)=(\langle R\rangle(t),\langle R_2\rangle(t),\cdots,\langle R_m(t)\rangle(t))^T$, $\boldsymbol{\alpha}=\alpha\,(1,1,\cdots,1)^T$ and $\boldsymbol{M}$ is an $m\times m$ triangular matrix whose diagonal is $(-1,-2,\cdots,-m)$. This implies that the system has a single fixed point and this is stable.

Now we can make m tend to infinity to conclude that the expectation and all the centered moments of R tend to an equilibrium that is obtained by solving

$$\begin{aligned}\tau_p\langle\dot{R}\rangle &= 0\\ \tau_p\langle\dot{R_n}\rangle &= 0\qquad\text{for } n\geq 2.\end{aligned}\tag{S26}$$

The solution can be expressed recursively as

$$\begin{aligned}\langle R\rangle &= \alpha\\ \langle R_n\rangle &= \frac{\alpha}{n}\left[1+\sum_{k=2}^{n-2}\binom{n}{k}\langle R_k\rangle\right]\qquad\text{for } n\geq 2.\end{aligned}\tag{S27}$$

1.4 Stationary distribution when α tends to infinity

We show now that the asymptotic distribution of R (i.e., the distribution of $R(t)$ when t goes to infinity) in the limit $\alpha \to \infty$ is nothing but a Gaussian distribution.

We denote the expectation and the nth centered moment of the asymptotic distribution by $\langle R \rangle$ and $\langle R_n \rangle$, respectively. These asymptotic moments are given by the recursion defined in Eq. (S27). For a given α, the asymptotic expectation and the variance of R are

$$\begin{array}{rcl} \langle R \rangle & = & \alpha \\ \langle R_2 \rangle & = & \alpha/2. \end{array} \tag{S28}$$

In order to characterize the asymptotic distribution, we consider its normalized version. To this end, for every t we define a new random variable $Z(t)$ by

$$Z(t) = \frac{R(t) - \langle R \rangle}{\sigma}, \tag{S29}$$

where $\sigma := \sqrt{\langle R_2 \rangle} = \sqrt{\alpha/2}$. The expectation and the centered moments of $Z(t)$ are

$$\langle Z \rangle(t) = \frac{\langle R \rangle(t) - \langle R \rangle}{\sigma}, \qquad \langle Z_n \rangle(t) = \frac{\langle R_n \rangle(t)}{\sigma^n} \qquad \text{for } n \geq 0. \tag{S30}$$

Denoting by $\langle Z \rangle$ and $\langle Z_n \rangle$ the expectation and the nth centered moment of $Z(t)$ in the limit $t \to \infty$, we have

$$\begin{array}{rcll} \langle Z \rangle & = & 0 & \\ \langle Z_2 \rangle & = & 1 & \\ \langle Z_n \rangle & = & \frac{\langle R_n \rangle}{\sigma^n}, & n \geq 3. \end{array} \tag{S31}$$

The goal is to prove that $Z(t)$ converges to a standard Gaussian distribution as $t \to \infty$ in the limit $\alpha \to \infty$. In particular, we will show that the moments of $Z(t)$ in this limit are the ones of a standard Gaussian distribution. The nth centered moment μ_n of a standard Gaussian distribution is

$$\mu_n = \begin{cases} 0 & \text{if } n \text{ is odd,} \\ (n-1)!! & \text{if } n \text{ is even,} \end{cases} \tag{S32}$$

where

$$n!! := \begin{cases} n(n-2)(n-4)\cdots 3 \cdot 1 & \text{if } n \text{ is odd} \\ n(n-2)(n-4)\cdots 4 \cdot 2 & \text{if } n \text{ is even.} \end{cases}$$

We should thus prove that $\lim_{\alpha \to \infty} \langle Z_n \rangle = \mu_n$ for $n \geq 3$. This is accomplished as follows. From Eqs. (S27), (S31) we get the following recursion for the centered moments of $Z(t)$ when $t \to \infty$:

$$\langle Z_n \rangle = \frac{\alpha}{n\sigma^n} \left(1 + \sum_{k=2}^{n-2} \binom{n}{k} \sigma^k \langle Z_k \rangle \right) \qquad n \geq 2. \tag{S33}$$

We reason by induction on n. Recall that, by definition, $\alpha = 2\sigma^2$, so we can express the limit $\alpha \to \infty$ as a limit $\sigma \to \infty$.

- For $n = 3$, $\langle Z_3 \rangle = \frac{\alpha}{3\sigma^3} = \frac{2}{3\sigma}$, so $\lim_{\sigma \to \infty} \langle Z_3 \rangle = 0$ as desired.
- For $n = 4$, $\langle Z_4 \rangle = \frac{\alpha}{4\sigma^4} \left(1 + \binom{4}{2} \sigma^2 \langle Z_2 \rangle \right) = \frac{1}{2\sigma^2} \left(1 + \binom{4}{2} \sigma^2 \right)$, so $\lim_{\sigma \to \infty} \langle Z_4 \rangle = \binom{4}{2}/2 = 3 = 3!!$ as desired.
- Let us assume that the result is true up to $n-1$. The limit of nth centered moment is

$$\begin{aligned} \lim_{\sigma \to \infty} \langle Z_n \rangle &= \lim_{\sigma \to \infty} \frac{\alpha}{n\sigma^n} \left(1 + \sum_{k=2}^{n-2} \binom{n}{k} \sigma^k \langle Z_k \rangle \right) \\ &= \lim_{\sigma \to \infty} \begin{cases} \frac{2\sigma^2}{n\sigma^n} \binom{n}{n-3} \sigma^{n-3} (n-4)!! & \text{if } n \text{ is odd} \\ \frac{2\sigma^2}{n\sigma^n} \binom{n}{n-2} \sigma^{n-2} (n-3)!! & \text{if } n \text{ is even} \end{cases} \\ &= \lim_{\sigma \to \infty} \begin{cases} \frac{2(n-4)!!}{n\sigma} \binom{n}{n-3} & \text{if } n \text{ is odd} \\ \frac{2(n-3)!!}{n} \binom{n}{n-2} & \text{if } n \text{ is even} \end{cases} \\ &= \begin{cases} 0 & \text{if } n \text{ is odd} \\ (n-1)!! & \text{if } n \text{ is even.} \end{cases} \end{aligned} \tag{S34}$$

We conclude that the result is true for n as well, so we have proved what we wanted.

The corollary of this is the following: for $\alpha = \tau_p \nu$ large enough, as $t \to \infty$, R approaches to a Gaussian distribution with mean α and variance $\alpha/2$.

2 INTEGRAL OF THE INPUT CURRENT

We suppose that the dynamics is on a stationary state so that the synaptic weights and the firing rates do not change in time. We take a neuron i and consider its recurrent input current at time t,

$$I_i^{\text{rec}}(t) = \sum_{j=1}^{N} a_{ij}\, w_{ij} \sum_k \delta(t - t_j^k - d_j). \tag{S35}$$

We also assume that the spike times of every neuron j in the network are stochastic and generated by a Poisson process of rate ν_j and that these Poisson processes are independent. The integral of the recurrent input between t and $t + \tau$, i.e.,

$$X_i^{\text{rec}}(t, \tau) := \int_t^{t+\tau} I_i^{\text{rec}}(s)\, \mathrm{d}s, \tag{S36}$$

is thus a stochastic variable. We want to compute its mean and variance assuming that we know what the stationary firing rates and the synaptic weights are. We rewrite $X_i^{\text{rec}}(t, \tau)$ as

$$\begin{aligned} X_i^{\text{rec}}(t, \tau) &= \sum_{j=1}^{N} a_{ij} w_{ij}\, Y_j(t, \tau) \\ Y_j(t, \tau) &:= \int_t^{t+\tau} \sum_k \delta(s - t_j^k - d_j)\, \mathrm{d}s, \end{aligned} \tag{S37}$$

so that

$$\begin{aligned} \mathbb{E}[X_i^{\text{rec}}(t, \tau)] &= \sum_{j=1}^{N} a_{ij} w_{ij}\, \mathbb{E}[Y_j(t, \tau)] \\ \mathrm{Var}[X_i^{\text{rec}}(t, \tau)] &= \sum_{j=1}^{N} a_{ij} w_{ij}^2\, \mathrm{Var}[Y_j(t, \tau)]. \end{aligned} \tag{S38}$$

Because of the definition of $Y_j(t, \tau)$ as an integral of the sum of Dirac delta distributions, it can be expressed simply as

$$Y_j(t, \tau) = \text{number of spikes emitted by neuron } j \text{ in } [t - d_j, t - d_j + \tau]. \tag{S39}$$

This means that, under the Poisson hypothesis,

$$Y_j(t, \tau) \sim \text{Poisson}(\tau \nu_j) \tag{S40}$$

so

$$\begin{aligned} \mathbb{E}[Y_j(t, \tau)] &= \tau \nu_j \\ \mathrm{Var}[Y_j(t, \tau)] &= \tau \nu_j. \end{aligned} \tag{S41}$$

We thus have

$$\begin{aligned} \mathbb{E}[X_i^{\text{rec}}(t, \tau)] &= \tau \sum_{j=1}^{N} a_{ij} w_{ij}\, \nu_j \\ \mathrm{Var}[X_i^{\text{rec}}(t, \tau)] &= \tau \sum_{j=1}^{N} a_{ij} w_{ij}^2 \nu_j. \end{aligned} \tag{S42}$$

We can now consider the total input current, which is the sum of the recurrent input $I_i^{\text{rec}}(t)$ and the external input, i.e.,

$$I_i^{\text{ext}}(t) = w_{\text{ext}} \sum_{j=1}^{K_{\text{ext}}} \sum_k \delta(t - t_{ij}^k). \tag{S43}$$

If the external spike trains are generated by independent Poisson processes of rate ν_{ext}, the integral of the total input current between t and $t + \tau$,

$$X_i(t, \tau) := \int_t^{t+\tau} \left(I_i^{\text{rec}}(s) + I_i^{\text{ext}}(s) \right) \mathrm{d}s, \tag{S44}$$

satisfies

$$\begin{aligned} \mathbb{E}[X_i(t, \tau)] &= \tau \left(\sum_{j=1}^{N} a_{ij} w_{ij}\, \nu_j + K_{\text{ext}} w_{\text{ext}} \nu_{\text{ext}} \right) \\ \mathrm{Var}[X_i(t, \tau)] &= \tau \left(\sum_{j=1}^{N} a_{ij} w_{ij}^2 \nu_j + K_{\text{ext}} w_{\text{ext}}^2 \nu_{\text{ext}} \right). \end{aligned} \tag{S45}$$

3 NOTES ON THE DEGREE DISTRIBUTION WHEN THERE IS A SINGLE NEURONAL TYPE

When the network is composed of only one type of neurons (either E or I), we assume that the binary structure of the connection network is specified via a joint in/out-degree distribution, given by a joint probability density function $\rho_{\mathrm{in,out}}$. This should be interpreted in the following way: the degrees of distinct neurons are independent random variables and the distribution of every pair of individual in/out-degrees is given by $\rho_{\mathrm{in,out}}$. There is no additional structure beyond this degree distribution, that is, given two neurons i and j such that the in-degree of i is k and the out-degree of j is l, the probability that they are connected is

$$P(i \leftarrow j \,|\, K_i^{\mathrm{in}} = k, K_j^{\mathrm{out}} = l) = \frac{kl}{N\langle K\rangle}, \tag{S46}$$

with

$$\langle K\rangle := \mathbb{E}[K^{\mathrm{in}}] = \mathbb{E}[K^{\mathrm{out}}]. \tag{S47}$$

In networks of this kind, the distribution of in- and out-degrees among connected neurons might be biased with respect to the distribution of the same degrees in the whole network, and these biases can be analytically computed. To do so, we consider an arbitrary pair of connected neurons, i and j $(i \neq j)$, where i is postsynaptic and j is presynaptic, i.e., $i \leftarrow j$.

3.1 In-degree of a postsynaptic neuron

We start by computing the distribution of the in-degree of i conditioned to the fact that it is postsynaptic to j. To do so, we first compute this distribution when we know what the out-degree of j is:

$$\begin{aligned} P(K_i^{\mathrm{in}} = k \,|\, i \leftarrow j, K_j^{\mathrm{out}} = l) &= \frac{P(i \leftarrow j \,|\, K_i^{\mathrm{in}} = k, K_j^{\mathrm{out}} = l) P(K_i^{\mathrm{in}} = k \,|\, K_j^{\mathrm{out}} = l)}{P(i \leftarrow j \,|\, K_j^{\mathrm{out}} = l)} \\ &= \frac{kl}{N\langle K\rangle} \frac{P(K_i^{\mathrm{in}} = k \,|\, K_j^{\mathrm{out}} = l)}{\sum_m P(i \leftarrow j \,|\, K_i^{\mathrm{in}} = m, K_j^{\mathrm{out}} = l) P(K_i^{\mathrm{in}} = m \,|\, K_j^{\mathrm{out}} = l)} \\ &= \frac{kl}{N\langle K\rangle} \frac{P(K_i^{\mathrm{in}} = k)}{\sum_m \frac{ml}{N\langle K\rangle} P(K_i^{\mathrm{in}} = m)} \\ &= \frac{k}{\langle K\rangle} P(K_i^{\mathrm{in}} = k), \end{aligned} \tag{S48}$$

which derives from Eq. (S46) and from the assumption that in/out-degrees are independent from neuron to neuron, i.e.,

$$P(K_i^{\mathrm{in}} = k \,|\, K_j^{\mathrm{out}} = l) = P(K_i^{\mathrm{in}} = k) \qquad \forall i \neq j. \tag{S49}$$

Eq. (S48) shows that the distribution of the in-degree of a postsynaptic neuron does not depend on the presynaptic neuron's degree:

$$P(K_i^{\mathrm{in}} = k \,|\, i \leftarrow j, K_j^{\mathrm{out}} = l) = P(K_i^{\mathrm{in}} = k \,|\, i \leftarrow j). \tag{S50}$$

It also shows that this distribution is biased with respect to the distribution of in-degrees in the network: in-degrees larger than the average value $\langle K\rangle$ are overrepresented and in-degrees smaller than the average are underrepresented. In particular, the expectation of the in-degree of a postsynaptic neuron is larger than the expectation of the in-degree of a random neuron:

$$\mathbb{E}[K_i^{\mathrm{in}} \,|\, i \leftarrow j] = \langle K\rangle + \frac{\mathrm{Var}(K^{\mathrm{in}})}{\langle K\rangle}. \tag{S51}$$

3.2 Out-degree of a presynaptic neuron

To characterize the distribution of the out-degree of a presynaptic neuron, we perform analogous computations and they give

$$P(K_j^{\mathrm{out}} = l \,|\, i \leftarrow j, K_i^{\mathrm{in}} = k) = \frac{l}{\langle K\rangle} P(K_j^{\mathrm{out}} = l) = P(K_j^{\mathrm{out}} = l \,|\, i \leftarrow j) \tag{S52}$$

and

$$\mathbb{E}[K_j^{\mathrm{out}} \,|\, i \leftarrow j] = \langle K\rangle + \frac{\mathrm{Var}(K^{\mathrm{out}})}{\langle K\rangle}. \tag{S53}$$

3.3 In-degree of a presynaptic neuron

Now we want to characterize the distribution of the in-degree of the presynaptic neuron j. We have

$$\begin{aligned} P(K_j^{\text{in}} = m \,|\, i \leftarrow j, K_i^{\text{in}} = k) &= \sum_l P(K_j^{\text{in}} = m \,|\, i \leftarrow j, K_i^{\text{in}} = k, K_j^{\text{out}} = l) P(K_j^{\text{out}} = l \,|\, i \leftarrow j, K_i^{\text{in}} = k) \\ &= \sum_l \frac{P(i \leftarrow j \,|\, K_j^{\text{in}} = m, K_i^{\text{in}} = k, K_j^{\text{out}} = l) P(K_j^{\text{in}} = m \,|\, K_i^{\text{in}} = k, K_j^{\text{out}} = l)}{P(i \leftarrow j \,|\, K_i^{\text{in}} = k, K_j^{\text{out}} = l)} P(K_j^{\text{out}} = l \,|\, i \leftarrow j, K_i^{\text{in}} = k) \\ &= \sum_l \frac{P(i \leftarrow j \,|\, K_i^{\text{in}} = k, K_j^{\text{out}} = l) P(K_j^{\text{in}} = m \,|\, K_j^{\text{out}} = l)}{P(i \leftarrow j \,|\, K_i^{\text{in}} = k, K_j^{\text{out}} = l)} \frac{l}{\langle K \rangle} P(K_j^{\text{out}} = l) \\ &= \frac{1}{\langle K \rangle} \sum_l l P(K_j^{\text{in}} = m \,|\, K_j^{\text{out}} = l) P(K_j^{\text{out}} = l) \\ &= \frac{1}{\langle K \rangle} \left(\sum_l l P(K_j^{\text{out}} = l \,|\, K_j^{\text{in}} = m) \right) P(K_j^{\text{in}} = m) \\ &= \frac{\mathbb{E}\left[K_j^{\text{out}} \,|\, K_j^{\text{in}} = m \right]}{\langle K \rangle} P(K_j^{\text{in}} = m), \end{aligned} \tag{S54}$$

where in the 3rd equality we used Eq. (S52). Again, this is independent of the in-degree of i. Since the conditional expectation $\mathbb{E}\left[K_j^{\text{out}} \,|\, K_j^{\text{in}} = m\right]$ is independent of the index j because the degree distribution imposed in the network is the same for all neurons, we can use the notation

$$\langle K^{\text{out}} \,|\, K^{\text{in}} = m \rangle := \mathbb{E}\left[K_j^{\text{out}} \,|\, K_j^{\text{in}} = m \right] \tag{S55}$$

and write

$$P(K_j^{\text{in}} = m \,|\, i \leftarrow j, K_i^{\text{in}} = k) = \frac{\langle K^{\text{out}} \,|\, K^{\text{in}} = m \rangle}{\langle K \rangle} P(K_j^{\text{in}} = m) = P(K_j^{\text{in}} = m \,|\, i \leftarrow j). \tag{S56}$$

Contrary to the out-degree of the presynaptic neuron j, which is always biased [see Eq. (S52)], the in-degree of j is only biased when there is a correlation (either positive or negative) between individual in/out-degrees. In the case of independent degrees, the conditional expectation on Eq. (S56) equals the expected degree $\langle K \rangle$ and the in-degree distribution is preserved.

3.4 The in-degrees of two connected neurons are independent random variables

An important observation derived from Eq. (S56) is that the in-degrees of two connected neurons are independent random variables. This is simply because

$$\begin{aligned} P(K_i^{\text{in}} = k, K_j^{\text{in}} = m \,|\, i \leftarrow j) &= P(K_j^{\text{in}} = m \,|\, i \leftarrow j, K_i^{\text{in}} = k) P(K_i^{\text{in}} = k \,|\, i \leftarrow j) \\ &= P(K_j^{\text{in}} = m \,|\, i \leftarrow j) P(K_i^{\text{in}} = k \,|\, i \leftarrow j), \end{aligned} \tag{S57}$$

where the last equality follows from Eq. (S56).

3.5 In-degree p.d.f. for a presynaptic neuron in two particular cases

Let us treat the degrees as if they were continuous variables. We denote by $\rho^{\text{pre}}_{K^{\text{in}}}$ the p.d.f. of the in-degrees among the presynaptic neurons to a given neuron. As it has been shown in the preceding section, $\rho^{\text{pre}}_{K^{\text{in}}}$ depends on the joint in/out-degree distribution in the network through

$$\rho^{\text{pre}}_{K^{\text{in}}}(k) = \frac{\langle K^{\text{out}} \,|\, K^{\text{in}} = k \rangle}{\langle K \rangle} \rho_{K^{\text{in}}}(k), \tag{S58}$$

where $\rho_{K^{\text{in}}}$ is the marginal p.d.f. of the in-degrees in the network. The conditional expectation in Eq. (S58) is computed as

$$\langle K^{\text{out}} \,|\, K^{\text{in}} = k \rangle = \int_0^\infty y \, \rho_{\text{out}|\text{in}}(y \,|\, k) \, \mathrm{d}y = \frac{1}{\rho_{K^{\text{in}}}(k)} \int_0^\infty y \, \rho_{\text{in,out}}(k, y) \, \mathrm{d}y, \tag{S59}$$

where $\rho_{\text{out}|\text{in}}$ is the p.d.f. of the out-degree conditioned to the in-degree and $\rho_{\text{in,out}}$ is the p.d.f. of the joint degree distribution.

Eqs. (S58), (S59) thus specify how to compute the biased density $\rho^{\text{pre}}_{K^{\text{in}}}$:

$$\rho^{\text{pre}}_{K^{\text{in}}}(k) = \frac{1}{\langle K \rangle} \int_0^\infty y \, \rho_{\text{in,out}}(k, y) \, \mathrm{d}y. \tag{S60}$$

Let $(K^{\text{in}}, K^{\text{out}})$ be the pair of in- and out-degrees of a random neuron in the network. We will compute $\rho^{\text{pre}}_{K^{\text{in}}}(k)$ in two particular cases.

1. Assume that

$$(K^{\text{in}}, K^{\text{out}}) \sim \text{Normal}(\boldsymbol{\mu}, \boldsymbol{\Sigma}) \tag{S61}$$

with

$$\boldsymbol{\mu} = (\langle K\rangle, \langle K\rangle)^T, \qquad \boldsymbol{\Sigma} = \begin{pmatrix} \sigma_{\text{in}}^2 & r\sigma_{\text{in}}\sigma_{\text{out}} \\ r\sigma_{\text{in}}\sigma_{\text{out}} & \sigma_{\text{out}}^2 \end{pmatrix}, \tag{S62}$$

r being the correlation coefficient between in- and out-degrees. From Eqs. (S61), (S62), it follows that the out-degree conditioned to the in-degree taking the value of k, $(K^{\text{out}}|K^{\text{in}} = k)$, is also normally distributed, with mean

$$\langle K^{\text{out}}|K^{\text{in}} = k\rangle = \langle K\rangle + r\frac{\sigma_{\text{out}}}{\sigma_{\text{in}}}(k - \langle K\rangle), \tag{S63}$$

so

$$\rho^{\text{pre}}_{K^{\text{in}}}(k) = \left[1 + r\frac{\sigma_{\text{out}}}{\sigma_{\text{in}}}\left(\frac{k}{\langle K\rangle} - 1\right)\right]\rho_{K^{\text{in}}}(k). \tag{S64}$$

2. Assume that $(K^{\text{in}}, K^{\text{out}})$ is constructed as follows:

$$\begin{aligned} K^{\text{in}} &= Z_1 + Z_2 \\ K^{\text{out}} &= Z_1 + Z_3, \end{aligned} \tag{S65}$$

where Z_1, Z_2, Z_3 are positive and independent random variables with p.d.f.s ρ_1, ρ_2, ρ_3. In this case,

$$\begin{aligned} \rho_{\text{in,out}}(x, y) &= \int_0^\infty \rho_{\text{in,out}}(x, y \,|\, Z_1 = z_1)\, \rho_1(z_1)\, \mathrm{d}z_1 \\ &= \int_0^\infty \rho_2(x - z_1)\rho_3(y - z_1)\rho_1(z_1)\, \mathrm{d}z_1. \end{aligned} \tag{S66}$$

Inserting (S66) in (S60) we obtain

$$\begin{aligned} \rho^{\text{pre}}_K(k) &= \frac{1}{\langle K\rangle}\int_0^\infty y \int_0^\infty \rho_2(k - z_1)\rho_3(y - z_1)\rho_1(z_1)\, \mathrm{d}z_1\, \mathrm{d}y \\ &= \frac{1}{\langle K\rangle}\int_0^\infty \rho_2(k - z_1)\rho_1(z_1)\left(\int_0^\infty y\, \rho_3(y - z_1)\, \mathrm{d}y\right)\mathrm{d}z_1 \\ &= \frac{1}{\langle K\rangle}\int_0^\infty \rho_2(k - z_1)\rho_1(z_1)\left(\langle Z_3\rangle + z_1\right)\mathrm{d}z_1 \\ &= \frac{\langle Z_3\rangle}{\langle K\rangle}\int_0^\infty \rho_2(k - z_1)\rho_1(z_1)\, \mathrm{d}z_1 + \frac{1}{\langle K\rangle}\int_0^\infty z_1\rho_2(k - z_1)\rho_1(z_1)\, \mathrm{d}z_1 \\ &= \frac{\langle Z_3\rangle}{\langle K\rangle}\int_0^\infty \rho_{K^{\text{in}},Z_1}(k, z_1)\, \mathrm{d}z_1 + \frac{1}{\langle K\rangle}\int_0^\infty z_1\rho_2(k - z_1)\rho_1(z_1)\, \mathrm{d}z_1 \\ &= \frac{\langle Z_3\rangle}{\langle K\rangle}\rho_{K^{\text{in}}}(k) + \frac{1}{\langle K\rangle}\int_0^\infty z_1\rho_2(k - z_1)\rho_1(z_1)\, \mathrm{d}z_1. \end{aligned} \tag{S67}$$

4 NOTES ON THE DEGREE DISTRIBUTION WHEN THERE ARE TWO NEURONAL TYPES

In the case of a network on N_E E neurons and N_I I neurons, every neuron is characterized by an E in-degree (that is, the in-degree from the E population), an E out-degree, an I in-degree, and an I out-degree. For simplicity we assume that the distributions of these degrees are the same regardless of whether the neuron belongs to the E or I population. The set of degrees associated to one neuron is also independent to the set of degrees of any other neuron. For any given neuron, we assume that the pair of degrees from/to population E is independent of the pair of degrees from/to population I. The two E (and I) degrees could, nevertheless, be correlated. Thus, the binary structure of the connection network is specified via two distinct joint p.d.f.s, $\rho^E_{\text{in,out}}$, $\rho^I_{\text{in,out}}$, that specify how these two degree pairs are distributed. There is no additional structure beyond the degrees: given a neuron i in population α and a neuron j in population β, with degrees

$$\begin{aligned} \boldsymbol{K_i} &= \left(K_i^{\text{E,in}}, K_i^{\text{E,out}}, K_i^{\text{I,in}}, K_i^{\text{I,out}}\right), \\ \boldsymbol{K_j} &= \left(K_j^{\text{E,in}}, K_j^{\text{E,out}}, K_j^{\text{I,in}}, K_j^{\text{I,out}}\right), \end{aligned} \tag{S68}$$

the probability that they are connected once these degrees are known is

$$P\left(i \leftarrow j \,|\, \boldsymbol{K_i}, \boldsymbol{K_j}, i \in \alpha, j \in \beta\right) = \frac{K_i^{\beta,\text{in}}K_j^{\alpha,\text{out}}}{C}, \tag{S69}$$

with

$$C = N_\alpha\, \langle K^{\beta,\text{in}}\rangle = N_\beta\, \langle K^{\alpha,\text{out}}\rangle. \tag{S70}$$

Notice that this in particular imposes a constraint on the degree expectations $\langle K^{\beta,\text{in}}\rangle$, $\langle K^{\alpha,\text{out}}\rangle$ for $\alpha, \beta \in \{E, I\}$.

From Eqs. (S69), (S70) it follows that

$$P\left(i \leftarrow j \,|\, i \in \alpha, j \in \beta\right) = \frac{\langle K^{\alpha,\mathrm{out}} \rangle}{N_\alpha} = \frac{\langle K^{\beta,\mathrm{in}} \rangle}{N_\beta}. \tag{S71}$$

Next we compute the degree distributions among connected neurons, which might be biased with respect to the original distributions. For this, we pick two connected neurons i, j, $i \leftarrow j$ (i.e., i is postsynaptic and j is presynaptic), with $i \in \alpha$, $j \in \beta$, $\alpha, \beta \in \{E, I\}$. Because we assume that the degrees from/to population E are independent of the degrees from/to population I, knowing that $i \leftarrow j$ with $j \in \beta$ can bias the degrees of i from/to population β but not those from/to the other population (and analogously for node j). We thus only compute the bias in the cases in which a bias may exist.

4.1 In-degree of a postsynaptic neuron

We have

$$\begin{aligned}
P\left(K_i^{\beta,\mathrm{in}} = k \,|\, i \leftarrow j, i \in \alpha, j \in \beta\right) &= \sum_l P\left(K_i^{\beta,\mathrm{in}} = k, K_j^{\alpha,\mathrm{out}} = l \,|\, i \leftarrow j, i \in \alpha, j \in \beta\right) \\
&= \sum_l \frac{P\left(i \leftarrow j \,|\, K_i^{\beta,\mathrm{in}} = k, K_j^{\alpha,\mathrm{out}} = l, i \in \alpha, j \in \beta\right) P\left(K_i^{\beta,\mathrm{in}} = k, K_j^{\alpha,\mathrm{out}} = l \,|\, i \in \alpha, j \in \beta\right)}{P\left(i \leftarrow j \,|\, i \in \alpha, j \in \beta\right)} \\
&= \frac{kP\left(K_i^{\beta,\mathrm{in}} = k\right)}{CP\left(i \leftarrow j \,|\, i \in \alpha, j \in \beta\right)} \sum_l lP\left(K_j^{\alpha,\mathrm{out}} = l\right) \\
&= \frac{k}{\langle K^{\beta,\mathrm{in}} \rangle} P\left(K_i^{\beta,\mathrm{in}} = k\right).
\end{aligned} \tag{S72}$$

4.2 Out-degree of a presynaptic neuron

Analogously,

$$P\left(K_j^{\alpha,\mathrm{out}} = l \,|\, i \leftarrow j, i \in \alpha, j \in \beta\right) = \frac{l}{\langle K^{\alpha,\mathrm{out}} \rangle} P(K_j^{\alpha,\mathrm{out}} = l). \tag{S73}$$

4.3 In-degree of a presynaptic neuron

Similarly,

$$\begin{aligned}
P\left(K_j^{\alpha,\mathrm{in}} = m \,|\, i \leftarrow j, i \in \alpha, j \in \beta\right) &= \sum_{k,l} P\left(K_j^{\alpha,\mathrm{in}} = m, K_j^{\alpha,\mathrm{out}} = l, K_i^{\beta,\mathrm{in}} = k \,|\, i \leftarrow j, i \in \alpha, j \in \beta\right) \\
&= \sum_{k,l} \frac{P\left(i \leftarrow j \,|\, K_j^{\alpha,\mathrm{in}} = m, K_j^{\alpha,\mathrm{out}} = l, K_i^{\beta,\mathrm{in}} = k, i \in \alpha, j \in \beta\right)}{P\left(i \leftarrow j \,|\, i \in \alpha, j \in \beta\right)} \\
&\quad \times P\left(K_j^{\alpha,\mathrm{in}} = m, K_j^{\alpha,\mathrm{out}} = l, K_i^{\beta,\mathrm{in}} = k \,|\, i \in \alpha, j \in \beta\right) \\
&= \frac{1}{CP\left(i \leftarrow j \,|\, i \in \alpha, j \in \beta\right)} \left(\sum_k kP(K_i^{\beta,\mathrm{in}} = k)\right) \left(\sum_l lP(K_j^{\alpha,\mathrm{in}} = m, K_j^{\alpha,\mathrm{out}} = l)\right) \\
&= \frac{1}{\langle K^{\alpha,\mathrm{out}} \rangle} \left(\sum_l lP(K_j^{\alpha,\mathrm{out}} = l \,|\, K_j^{\alpha,\mathrm{in}} = m)\right) P(K_j^{\alpha,\mathrm{in}} = m) \\
&= \frac{\langle K^{\alpha,\mathrm{out}} \,|\, K^{\alpha,\mathrm{in}} = m \rangle}{\langle K^{\alpha,\mathrm{out}} \rangle} P\left(K_j^{\alpha,\mathrm{in}} = m\right).
\end{aligned} \tag{S74}$$

Interpreting the degrees as continuous variables, and denoting the marginal p.d.f. of the in-degree from population α of a random neuron as ρ_K^α, we can write the previous equation for the in-degree from population α of a presynaptic neuron to a neuron in α as

$$\rho_K^{\mathrm{pre},\alpha}(m) = \frac{\langle K^{\alpha,\mathrm{out}} \,|\, K^{\alpha,\mathrm{in}} = m \rangle}{\langle K^{\alpha,\mathrm{out}} \rangle} \rho_K^\alpha\left(m\right). \tag{S75}$$

5 MEAN-FIELD THEORY FOR NETWORKS WITH EXCITATORY AND INHIBITORY NEURONS

Here we outline the extension of the mean-field equations presented in the main text to a network composed of excitatory (E) and inhibitory (I) neurons. As pointed out before, throughout the text we call *E in-degree* the in-degree of one neuron that comes from the E population. The *E out-degree* is the out-degree that goes to the E population. We define analogously the I in- and out-degree. Also, an *E synaptic weight* is a weight that originates from an E (presynaptic) neuron, and an *I synaptic weight* originates from an I neuron.

We assume that the number of E/I incoming connections and the magnitude of the incoming E/I synaptic weights are statistically the same for both types of neurons. Interestingly, the fact that these statistics are the same for both neuronal types does not necessarily imply that the total input received is statistically the same. The reason is that correlations between individual in- and out-degrees bias the distribution of in-degrees among presynaptic neurons. The bias affects the E in-degree of the presynaptic neuron when the postsynaptic neuron is E and the I in-degree of the presynaptic neuron when the postsynaptic neuron is I [see

Eq. (S74)]. Since firing rates directly depend on in-degrees, the distribution of presynaptic firing rates is affected, and the rate bias is different depending on whether the postsynaptic neuron is E or I. However, as long as individual in/out-degrees are independent, no biases exist in the in-degrees of presynaptic neurons and the total input received is independent of the postsynaptic neuronal type. This greatly simplifies the dimension of the mean-field parameters and equations as we will show next.

As in the main text, we analyze different model scenarios separately.

5.1 Network with equivalent neurons

Suppose that any given neuron receives input from exactly K^E excitatory neurons and K^I inhibitory neurons. Also, in model A, the E synaptic weights are all the same. The I weights are all the same and equal in magnitude to the E ones but with opposite sign. In model B, the absolute value of E and I weights evolves in time according to the same form of plasticity rule.

This setting gives rise to a stationary state in which the firing rate ν is the same for all neurons, regardless of whether they are E or I. Let $w_E = w$ and $w_I = -w$ be the values of E and I synaptic weights, respectively. In model A, w is a parameter of the system, whereas in model B it is a function of the stationary rate: $w = w(\nu)$.

The quantities μ_i and σ_i of Eq. (**??**) in this case do not depend on i nor on the neuron type and read

$$\begin{aligned}\mu(\nu) &= \tau\left(K^E w\nu - K^I w\nu + K_{\text{ext}} w_{\text{ext}} \nu_{\text{ext}}\right)\\ \sigma^2(\nu) &= \tau\left(K^E w^2\nu + K^I w^2\nu + K_{\text{ext}} w_{\text{ext}}^2 \nu_{\text{ext}}\right)\end{aligned} \tag{S76}$$

in model A and

$$\begin{aligned}\mu(\nu) &= \tau\left(K^E w(\nu)\nu - K^I w(\nu)\nu + K_{\text{ext}} w_{\text{ext}} \nu_{\text{ext}}\right)\\ \sigma^2(\nu) &= \tau\left(K^E w(\nu)^2\nu + K^I w(\nu)^2\nu + K_{\text{ext}} w_{\text{ext}}^2 \nu_{\text{ext}}\right)\end{aligned} \tag{S77}$$

in model B. The stationary firing rate ν is found by solving

$$\nu = \phi\left(\mu(\nu), \sigma(\nu)\right). \tag{S78}$$

5.2 Heterogeneous network with no plasticity (model A)

In this case the network structure is the one defined in Section 4. The E synaptic weights are generated independently from a chosen weight distribution and are constant in time. The I weights have the same structure but with negative sign.

Let us take a neuron i from population $\alpha \in \{E, I\}$. We write $\mu_i = \mu_{\alpha,i}$ and $\sigma_i = \sigma^2_{\alpha,i}$, with

$$\begin{aligned}\mu_{\alpha,i} &= \tau\left(S^{\alpha E}_{\mu,i} + S^{\alpha I}_{\mu,i} + K_{\text{ext}} w_{\text{ext}} \nu_{\text{ext}}\right)\\ \sigma^2_{\alpha,i} &= \tau\left(S^{\alpha E}_{\sigma,i} + S^{\alpha I}_{\sigma,i} + K_{\text{ext}} w^2_{\text{ext}} \nu_{\text{ext}}\right)\end{aligned} \tag{S79}$$

and

$$\begin{aligned}S^{\alpha E}_{\mu,i} &:= \sum_{j=1}^{K_i^E} w_{ij}\nu_j, \quad & S^{\alpha I}_{\mu,i} &:= \sum_{j=1}^{K_i^I} w_{ij}\nu_j\\ S^{\alpha E}_{\sigma,i} &:= \sum_{j=1}^{K_i^E} w^2_{ij}\nu_j, \quad & S^{\alpha I}_{\sigma,i} &:= \sum_{j=1}^{K_i^I} w^2_{ij}\nu_j,\end{aligned} \tag{S80}$$

where K_i^E and K_i^I are the excitatory and inhibitory in-degrees of neuron i. Notice that in the previous expressions the index j runs over the E incoming neighbors (in $S^{\alpha E}_{\mu,i}$ and $S^{\alpha E}_{\sigma,i}$) or over the I incoming neighbors (in $S^{\alpha I}_{\mu,i}$ and $S^{\alpha I}_{\sigma,i}$), so that for a fixed j, w_{ij} and ν_j are not the same on $S^{\alpha E}_{\mu,i}$ and on $S^{\alpha I}_{\mu,i}$, for example. This is why a pair of indexes $\alpha\beta$ is used in S_μ and S_σ, which specifies the pre- and the postsynaptic types involved in each case.

To deal with these sums, we rewrite Eq. (S80) as

$$\boldsymbol{S}_i^{\alpha\beta} := \begin{pmatrix} S^{\alpha\beta}_{\mu,i} \\ S^{\alpha\beta}_{\sigma,i}\end{pmatrix} = \sum_{j=1}^{K_i^\beta} \begin{pmatrix} w_{ij}\nu_j \\ w^2_{ij}\nu_j \end{pmatrix}, \qquad \beta \in \{E, I\}. \tag{S81}$$

Let

$$\boldsymbol{m}^{\alpha\beta} = \begin{pmatrix} m^{\alpha\beta}_\mu \\ m^{\alpha\beta}_\sigma \end{pmatrix}, \qquad \boldsymbol{\Sigma}^{\alpha\beta} = \begin{pmatrix} s^{2,\alpha\beta}_\mu & c^{\alpha\beta}_{\mu\sigma} \\ c^{\alpha\beta}_{\mu\sigma} & s^{2,\alpha\beta}_\sigma \end{pmatrix}, \qquad \beta \in \{E, I\}, \tag{S82}$$

be the mean vector and the covariance matrix of the elements in $S_i^{\alpha\beta}$, that is,

$$\begin{aligned} m^{\alpha\beta}_\mu &:= \mathbb{E}[\, w_{ij}\nu_j \,|\, j \to i,\, i \in \alpha, j \in \beta]\\ s^{2,\alpha\beta}_\mu &:= \mathrm{Var}[\, w_{ij}\nu_j \,|\, j \to i,\, i \in \alpha, j \in \beta]\\ m^{\alpha\beta}_\sigma &:= \mathbb{E}[\, w^2_{ij}\nu_j \,|\, j \to i,\, i \in \alpha, j \in \beta]\\ s^{2,\alpha\beta}_\sigma &:= \mathrm{Var}[\, w^2_{ij}\nu_j \,|\, j \to i,\, i \in \alpha, j \in \beta]\\ c^{\alpha\beta}_{\mu\sigma} &:= \mathrm{Cov}[\, w_{ij}\nu_j, w^2_{ij}\nu_j \,|\, i \in \alpha, j \to i,\, j \in \beta]. \end{aligned} \tag{S83}$$

Analogous arguments as the ones presented in the main text allow us to apply the Central Limit Theorem to the sums of Eq. (S81). Once the degrees are known, if they are large enough, the vector $\boldsymbol{S}_i^{\alpha\beta}$ is approximately distributed as a bivariate normal

vector with mean vector $K_i^\beta\, \boldsymbol{m}^{\alpha\beta}$ and covariance matrix $K_i^\beta \boldsymbol{\Sigma}^{\alpha\beta}$:

$$\boldsymbol{S}_i^{\alpha\beta} \quad = \quad \begin{pmatrix} S_{\mu,i}^{\alpha\beta} \\ S_{\sigma,i}^{\alpha\beta} \end{pmatrix} \quad = \quad K_i^\beta \begin{pmatrix} m_\mu^{\alpha\beta} \\ m_\sigma^{\alpha\beta} \end{pmatrix} + \sqrt{K_i^\beta} \begin{pmatrix} Y_i^{\alpha\beta} \\ Z_i^{\alpha\beta} \end{pmatrix}, \tag{S84}$$

where

$$\begin{pmatrix} Y_i^{\alpha\beta} \\ Z_i^{\alpha\beta} \end{pmatrix} \quad \sim \quad \mathcal{N}\left(\mathbf{0}, \boldsymbol{\Sigma}^{\alpha\beta}\right). \tag{S85}$$

We denote by $m_{\alpha\beta}$ and $s_{\alpha\beta}^2$ the mean and variance of the rate of an arbitrary neuron in population β which is presynaptic to a neuron in population α:

$$\begin{aligned} m_{\alpha\beta} &:= \quad \mathbb{E}[\nu_j \mid j \in \beta \text{ is presynaptic to a neuron in } \alpha], \\ s_{\alpha\beta}^2 &:= \quad \mathrm{Var}[\nu_j \mid j \in \beta \text{ is presynaptic to a neuron in } \alpha]. \end{aligned} \tag{S86}$$

Let $\boldsymbol{\theta} = (m_{\alpha\beta}, s_{\alpha\beta}^2)_{\alpha,\beta\in\{E,I\}}$ be the set of eight parameters defined previously. As in the case with a single population that we analyzed in the main text, the moments defined in Eq. (S83) for E synapses are expressed as a function of the moments of the excitatory weight distribution and the set of rate statistics $\boldsymbol{\theta}$ as

$$\begin{aligned} m_\mu^{\alpha E}(\boldsymbol{\theta}) &= \mathbb{E}[w]\, m_{\alpha E} \\ s_\mu^{2,\alpha E}(\boldsymbol{\theta}) &= \mathbb{E}[w^2]\, s_{\alpha E}^2 + \mathrm{Var}[w]\, m_{\alpha E}^2 \\ m_\sigma^{\alpha E}(\boldsymbol{\theta}) &= \mathbb{E}[w^2]\, m_{\alpha E} \\ s_\sigma^{2,\alpha E}(\boldsymbol{\theta}) &= \mathbb{E}[w^4]\, s_{\alpha E}^2 + \mathrm{Var}[w^2]\, m_{\alpha E}^2 \\ c_{\mu\sigma}^{\alpha E}(\boldsymbol{\theta}) &= \mathbb{E}[w^3]\, s_{\alpha E}^2 + \left(\mathbb{E}[w^3] - \mathbb{E}[w]\,\mathbb{E}[w^2]\right)\, m_{\alpha E}^2. \end{aligned} \tag{S87}$$

Since the inhibitory weights follow the same distribution in magnitude but have opposite sign, the moments for I synapses are

$$\begin{aligned} m_\mu^{\alpha I}(\boldsymbol{\theta}) &= -\mathbb{E}[w]\, m_{\alpha I} \\ s_\mu^{2,\alpha I}(\boldsymbol{\theta}) &= \mathbb{E}[w^2]\, s_{\alpha I}^2 + \mathrm{Var}[w]\, m_{\alpha I}^2 \\ m_\sigma^{\alpha I}(\boldsymbol{\theta}) &= \mathbb{E}[w^2]\, m_{\alpha I} \\ s_\sigma^{2,\alpha I}(\boldsymbol{\theta}) &= \mathbb{E}[w^4]\, s_{\alpha I}^2 + \mathrm{Var}[w^2]\, m_{\alpha I}^2 \\ c_{\mu\sigma}^{\alpha I}(\boldsymbol{\theta}) &= -\mathbb{E}[w^3]\, s_{\alpha I}^2 - \left(\mathbb{E}[w^3] - \mathbb{E}[w]\,\mathbb{E}[w^2]\right)\, m_{\alpha I}^2. \end{aligned} \tag{S88}$$

The firing rate ν_i of a neuron $i \in \alpha$ is therefore specified by the set of eight mean-field parameters $\boldsymbol{\theta}$ and by six identity variables associated to that neuron, $\boldsymbol{X}_i^\alpha = (K_i^E, Y_i^{\alpha E}, Z_i^{\alpha E}, K_i^I, Y_i^{\alpha I}, Z_i^{\alpha I})$ (whose distribution in turn depends on the mean-field parameters):

$$\nu_i = \nu_\alpha(\boldsymbol{\theta}, \boldsymbol{X}_i^\alpha) = \phi\left(\mu_\alpha\left(\boldsymbol{\theta}, \boldsymbol{X}_i^\alpha\right), \sigma_\alpha\left(\boldsymbol{\theta}, \boldsymbol{X}_i^\alpha\right)\right), \tag{S89a}$$

$$\begin{aligned} \mu_\alpha\left(\boldsymbol{\theta}, \boldsymbol{X}_i^\alpha\right) &= \tau\left(K_i^E\, m_\mu^{\alpha E}(\boldsymbol{\theta}) + \sqrt{K_i^E} Y_i^{\alpha E} + K_i^I\, m_\mu^{\alpha I}(\boldsymbol{\theta}) + \sqrt{K_i^I} Y_i^{\alpha I} + K_{\mathrm{ext}} w_{\mathrm{ext}} \nu_{\mathrm{ext}}\right) \\ \sigma_\alpha^2\left(\boldsymbol{\theta}, \boldsymbol{X}_i^\alpha\right) &= \tau\left(K_i^E\, m_\sigma^{\alpha E}(\boldsymbol{\theta}) + \sqrt{K_i^E} Z_i^{\alpha E} + K_i^I\, m_\sigma^{\alpha I}(\boldsymbol{\theta}) + \sqrt{K_i^I} Z_i^{\alpha I} + K_{\mathrm{ext}} w_{\mathrm{ext}}^2 \nu_{\mathrm{ext}}\right). \end{aligned} \tag{S89b}$$

The variables K_i^E, K_i^I are distributed according to the excitatory and inhibitory in-degree distribution imposed in the network and $(Y_i^{\alpha E}, Z_i^{\alpha E})$, $(Y_i^{\alpha I}, Z_i^{\alpha I})$ are normal bivariate independent vectors with zero mean and covariance matrices $\boldsymbol{\Sigma}^{\alpha E} = \boldsymbol{\Sigma}^{\alpha E}(\boldsymbol{\theta})$, $\boldsymbol{\Sigma}^{\alpha I} = \boldsymbol{\Sigma}^{\alpha I}(\boldsymbol{\theta})$, respectively. For all $\beta \in \{E, I\}$, the vector $(Y_i^{\alpha\beta}, Z_i^{\alpha\beta})$ is independent of K_i^E and K_i^I, and the identity vectors of all the neurons within population α, $\boldsymbol{X}_1^\alpha, \cdots, \boldsymbol{X}_{N_\alpha}^\alpha$, are i.i.d. The whole rate distribution in the network can be thus reconstructed from the set of eight statistics $\boldsymbol{\theta}$. These statistics fulfill

$$\begin{aligned} m_{\alpha\beta} &= \int_0^\infty \int_{-\infty}^\infty \int_{-\infty}^\infty \int_0^\infty \int_{-\infty}^\infty \int_{-\infty}^\infty \nu_\beta(\boldsymbol{\theta}, \boldsymbol{x})\, \rho_{\boldsymbol{X}}^{\boldsymbol{\theta},\alpha\beta}(\boldsymbol{x})\, \mathrm{d}\boldsymbol{x} &&=: \quad F_m^{\alpha\beta}(\boldsymbol{\theta}) \\ s_{\alpha\beta}^2 &= \int_0^\infty \int_{-\infty}^\infty \int_{-\infty}^\infty \int_0^\infty \int_{-\infty}^\infty \int_{-\infty}^\infty \left(\nu_\beta(\boldsymbol{\theta}, \boldsymbol{x}) - m_{\alpha\beta}\right)^2 \rho_{\boldsymbol{X}}^{\boldsymbol{\theta},\alpha\beta}(\boldsymbol{x})\, \mathrm{d}\boldsymbol{x} &&=: \quad F_{s^2}^{\alpha\beta}(\boldsymbol{\theta}), \qquad \alpha, \beta \in \{E, I\}, \end{aligned} \tag{S90}$$

where $\boldsymbol{x} = (k^E, y^E, z^E, k^I, y^I, z^I)$ and $\rho_{\boldsymbol{X}}^{\boldsymbol{\theta},\alpha\beta}(\boldsymbol{x})$ is the p.d.f. of $\boldsymbol{X}_j^\beta = (K_j^E, Y_j^{\beta E}, Z_j^{\beta E}, K_j^I, Y_j^{\beta I}, Z_j^{\beta I})$ for a neuron $j \in \beta$ that is presynaptic to a neuron in α:

$$\rho_{\boldsymbol{X}}^{\boldsymbol{\theta},\alpha\beta}(\boldsymbol{x}) = \rho_K^{\mathrm{pre},\alpha}(k^\alpha)\, \rho_K^{\bar{\alpha}}(k^{\bar{\alpha}})\, \rho_{Y,Z}^{\boldsymbol{\theta},\beta E}(y^E, z^E)\, \rho_{Y,Z}^{\boldsymbol{\theta},\beta I}(y^I, z^I), \tag{S91}$$

with

$$\bar{\alpha} = \begin{cases} I & \text{if } \alpha = E \\ E & \text{if } \alpha = I \end{cases} \tag{S92}$$

and $\rho_K^{\mathrm{pre},\alpha}$ being the p.d.f. of the in-degree from population α of a neuron that is presynaptic to a neuron in α (see section 4 for details), ρ_K^α being the p.d.f. of the in-degree from population α of a random neuron, $\rho_{Y,Z}^{\boldsymbol{\theta},\beta\gamma}$ being the p.d.f. of a normal bivariate

vector with mean $\mathbf{0}$ and covariance matrix $\mathbf{\Sigma}^{\beta\gamma}(\boldsymbol{\theta})$, $\gamma \in \{E, I\}$. The mean-field parameters in $\boldsymbol{\theta}$ are found by solving the system of eight unknowns and eight equations

$$\boldsymbol{\theta} = F(\boldsymbol{\theta}), \tag{S93}$$

with $F(\boldsymbol{\theta}) := \left(F_m^{EE}, F_{s^2}^{EE}, F_m^{EI}, F_{s^2}^{EI}, F_m^{IE}, F_{s^2}^{IE}, F_m^{II}, F_{s^2}^{II}\right)(\boldsymbol{\theta})$ and $F_m^{\alpha\beta}, F_{s^2}^{\alpha\beta}$ being the functions defined in Eq. (S90).

In the particular case in which individual E and I in/out-degrees are not correlated, the in-degree distributions among presynaptic neurons are not biased compared to the in-degree distributions in the network. As a consequence, the firing rates of presynaptic neurons are not biased either, and this makes the moments of Eq. (S86) be independent of the condition "j is presynaptic to a neuron in α". Thus, the moments in Eqs. (S87), (S88) are independent of α and so are the vectors $(Y_i^{\alpha\beta}, Z_i^{\alpha\beta})$ and $\boldsymbol{S}_i^{\alpha\beta}$ for $\beta \in \{E, I\}$. The result is that the quantities μ_α and σ_α^2 of Eq. (S89) are independent of α too: they are the same regardless of the population to which the postsynaptic neuron belongs. The final outcome is that the moments of Eq. (S86) are in fact independent of β as well. This means that the mean-field parameter set is just $\boldsymbol{\theta} = (m, s^2)$, with

$$\begin{aligned} m &:= \mathbb{E}[\nu_j], \\ s^2 &:= \mathrm{Var}[\nu_j]. \end{aligned} \tag{S94}$$

The firing rate of an arbitrary neuron i depends on its set of identity variables $\boldsymbol{X}_i = (K_i^E, Y_i^E, Z_i^E, K_i^I, Y_i^I, Z_i^I)$ through

$$\nu_i = \nu(\boldsymbol{\theta}, \boldsymbol{X}_i) = \phi\left(\mu\left(\boldsymbol{\theta}, \boldsymbol{X}_i\right), \sigma\left(\boldsymbol{\theta}, \boldsymbol{X}_i\right)\right), \tag{S95a}$$

$$\begin{aligned} \mu\left(\boldsymbol{\theta}, \boldsymbol{X}_i\right) &= \tau\left(K_i^E\, m_\mu^E(\boldsymbol{\theta}) + \sqrt{K_i^E} Y_i^E + K_i^I\, m_\mu^I(\boldsymbol{\theta}) + \sqrt{K_i^I} Y_i^I + K_{\text{ext}} w_{\text{ext}} \nu_{\text{ext}}\right) \\ \sigma^2\left(\boldsymbol{\theta}, \boldsymbol{X}_i\right) &= \tau\left(K_i^E\, m_\sigma^E(\boldsymbol{\theta}) + \sqrt{K_i^E} Z_i^E + K_i^I\, m_\sigma^I(\boldsymbol{\theta}) + \sqrt{K_i^I} Z_i^I + K_{\text{ext}} w_{\text{ext}}^2 \nu_{\text{ext}}\right), \end{aligned} \tag{S95b}$$

where

$$\begin{aligned} m_\mu^E(\boldsymbol{\theta}) &= \mathbb{E}[w]\, m \\ s_\mu^{2,E}(\boldsymbol{\theta}) &= \mathbb{E}[w^2]\, s^2 + \mathrm{Var}[w]\, m^2 \\ m_\sigma^E(\boldsymbol{\theta}) &= \mathbb{E}[w^2]\, m \\ s_\sigma^{2,E}(\boldsymbol{\theta}) &= \mathbb{E}[w^4]\, s^2 + \mathrm{Var}[w^2]\, m^2 \\ c_{\mu\sigma}^E(\boldsymbol{\theta}) &= \mathbb{E}[w^3]\, s^2 + \left(\mathbb{E}[w^3] - \mathbb{E}[w]\,\mathbb{E}[w^2]\right)\, m^2 \end{aligned} \tag{S96}$$

and

$$\begin{aligned} m_\mu^I(\boldsymbol{\theta}) &= -m_\mu^E(\boldsymbol{\theta}) \\ s_\mu^{2,I}(\boldsymbol{\theta}) &= s_\mu^{2,E}(\boldsymbol{\theta}) \\ m_\sigma^I(\boldsymbol{\theta}) &= m_\sigma^E(\boldsymbol{\theta}) \\ s_\sigma^{2,I}(\boldsymbol{\theta}) &= s_\sigma^{2,E}(\boldsymbol{\theta}) \\ c_{\mu\sigma}^I(\boldsymbol{\theta}) &= -c_{\mu\sigma}^E(\boldsymbol{\theta}). \end{aligned} \tag{S97}$$

The mean-field parameters fulfill

$$\begin{aligned} m &= \int_0^\infty \int_{-\infty}^\infty \int_{-\infty}^\infty \int_0^\infty \int_{-\infty}^\infty \int_{-\infty}^\infty \nu(\boldsymbol{\theta}, \boldsymbol{x})\, \rho_{\boldsymbol{X}}^{\boldsymbol{\theta}}(\boldsymbol{x})\, \mathrm{d}\boldsymbol{x} &&=: F_m(\boldsymbol{\theta}) \\ s^2 &= \int_0^\infty \int_{-\infty}^\infty \int_{-\infty}^\infty \int_0^\infty \int_{-\infty}^\infty \int_{-\infty}^\infty \left(\nu(\boldsymbol{\theta}, \boldsymbol{x}) - m\right)^2 \rho_{\boldsymbol{X}}^{\boldsymbol{\theta}}(\boldsymbol{x})\, \mathrm{d}\boldsymbol{x} &&=: F_{s^2}(\boldsymbol{\theta}), \end{aligned} \tag{S98}$$

where $\boldsymbol{x} = (k^E, y^E, z^E, k^I, y^I, z^I)$ and $\rho_{\boldsymbol{X}}^{\boldsymbol{\theta}}(\boldsymbol{x})$ is the p.d.f. of $\boldsymbol{X}_i = (K_i^E, Y_i^E, Z_i^E, K_i^I, Y_i^I, Z_i^I)$ for a random neuron i:

$$\rho_{\boldsymbol{X}}^{\boldsymbol{\theta}}(\boldsymbol{x}) = \rho_K^E(k^E)\, \rho_K^I(k^I)\, \rho_{Y,Z}^{\boldsymbol{\theta},E}(y^E, z^E)\, \rho_{Y,Z}^{\boldsymbol{\theta},I}(y^I, z^I), \tag{S99}$$

with ρ_K^α being the p.d.f. of the in-degree from population α of a random neuron, and $\rho_{Y,Z}^{\boldsymbol{\theta},\alpha}$ being the p.d.f. of a normal bivariate vector with mean $\mathbf{0}$ and covariance matrix $\mathbf{\Sigma}^\alpha(\boldsymbol{\theta})$, $\alpha \in \{E, I\}$.

This shows that the absence of degree correlations reduces the dimension of the mean-field equations from 8 to only 2.

5.3 Heterogeneous network with plastic synaptic weights (model B)

Now the binary interaction network is defined as in the previous section. Weights are plastic, and we assume that in the stationary state they are related to pre- and postsynaptic firing rates through

$$\begin{aligned} w_{ij} &= g^{\text{pre}}(\nu_j)\, g^{\text{post}}(\nu_i) &&\text{if } j \in E \\ w_{ij} &= -g^{\text{pre}}(\nu_j)\, g^{\text{post}}(\nu_i) &&\text{if } j \in I \end{aligned} \tag{S100}$$

for arbitrary functions g^{pre}, g^{post}. We take the plasticity rule to be the same in magnitude for all synapses to simplify the resulting equations.

The quantities $\mu_{\alpha,i}$ and $\sigma^2_{\alpha,i}$ of a neuron $i \in \alpha$ are now

$$\begin{aligned}
\mu_{\alpha,i} &= \tau\left(g^{\text{post}}(\nu_i)\sum_{j=1}^{K_i^E} g^{\text{pre}}(\nu_j)\nu_j - g^{\text{post}}(\nu_i)\sum_{j=1}^{K_i^I} g^{\text{pre}}(\nu_j)\nu_j + K_{\text{ext}}w_{\text{ext}}\nu_{\text{ext}}\right) \\
&= \tau\left(g^{\text{post}}(\nu_i)\left(S^{\alpha E}_{\mu,i} - S^{\alpha I}_{\mu,i}\right) + K_{\text{ext}}w_{\text{ext}}\nu_{\text{ext}}\right) \\
\sigma^2_{\alpha,i} &= \tau\left(g^{\text{post}}(\nu_i)^2\sum_{j=1}^{K_i^E} g^{\text{pre}}(\nu_j)^2\nu_j + g^{\text{post}}(\nu_i)^2\sum_{j=1}^{K_i^I} g^{\text{pre}}(\nu_j)^2\nu_j + K_{\text{ext}}w^2_{\text{ext}}\nu_{\text{ext}}\right) \\
&= \tau\left(g^{\text{post}}(\nu_i)^2\left(S^{\alpha E}_{\sigma,i} + S^{\alpha I}_{\sigma,i}\right) + K_{\text{ext}}w^2_{\text{ext}}\nu_{\text{ext}}\right),
\end{aligned} \tag{S101}$$

with

$$\begin{aligned}
S^{\alpha E}_{\mu,i} &:= \sum_{j=1}^{K_i^E} g^{\text{pre}}(\nu_j)\nu_j, \quad & S^{\alpha I}_{\mu,i} &:= \sum_{j=1}^{K_i^I} g^{\text{pre}}(\nu_j)\nu_j, \\
S^{\alpha E}_{\sigma,i} &:= \sum_{j=1}^{K_i^E} g^{\text{pre}}(\nu_j)^2\nu_j, \quad & S^{\alpha I}_{\sigma,i} &:= \sum_{j=1}^{K_i^I} g^{\text{pre}}(\nu_j)^2\nu_j,
\end{aligned} \tag{S102}$$

where, as before, the jth element in the sums over E neurons is different from the jth element in the sums over I neurons. Again, once the degrees are known, the sum

$$\boldsymbol{S}_i^{\alpha\beta} := \begin{pmatrix} S^{\alpha\beta}_{\mu,i} \\ S^{\alpha\beta}_{\sigma,i} \end{pmatrix} = \sum_{j=1}^{K_i^\beta} \begin{pmatrix} g^{\text{pre}}(\nu_j)\nu_j \\ g^{\text{pre}}(\nu_j)^2\nu_j \end{pmatrix}, \qquad \beta \in \{E, I\}, \tag{S103}$$

can be assumed to follow a normal distribution with mean vector $K_i^\beta \boldsymbol{m}^{\alpha\beta}$ and covariance matrix $K_i^\beta \boldsymbol{\Sigma}^{\alpha\beta}$:

$$\boldsymbol{S}_i^{\alpha\beta} = K_i^\beta \begin{pmatrix} m_\mu^{\alpha\beta} \\ m_\sigma^{\alpha\beta} \end{pmatrix} + \sqrt{K_i^\beta}\begin{pmatrix} Y_i^{\alpha\beta} \\ Z_i^{\alpha\beta} \end{pmatrix}, \tag{S104}$$

where

$$\begin{pmatrix} Y_i^{\alpha\beta} \\ Z_i^{\alpha\beta} \end{pmatrix} \sim \mathcal{N}\left(\boldsymbol{0}, \boldsymbol{\Sigma}^{\alpha\beta}\right) \tag{S105}$$

and

$$\boldsymbol{m}^{\alpha\beta} = \begin{pmatrix} m_\mu^{\alpha\beta} \\ m_\sigma^{\alpha\beta} \end{pmatrix}, \qquad \boldsymbol{\Sigma}^{\alpha\beta} = \begin{pmatrix} s_\mu^{2,\alpha\beta} & c_{\mu\sigma}^{\alpha\beta} \\ c_{\mu\sigma}^{\alpha\beta} & s_\sigma^{2,\alpha\beta} \end{pmatrix}, \tag{S106}$$

$$\begin{aligned}
m_\mu^{\alpha\beta} &:= \mathbb{E}[\, g^{\text{pre}}(\nu_j)\nu_j \,|\, j \to i,\, i \in \alpha, j \in \beta] \\
s_\mu^{2,\alpha\beta} &:= \text{Var}[\, g^{\text{pre}}(\nu_j)\nu_j \,|\, j \to i,\, i \in \alpha, j \in \beta] \\
m_\sigma^{\alpha\beta} &:= \mathbb{E}[\, g^{\text{pre}}(\nu_j)^2\nu_j \,|\, j \to i,\, i \in \alpha, j \in \beta] \\
s_\sigma^{2,\alpha\beta} &:= \text{Var}[\, g^{\text{pre}}(\nu_j)^2\nu_j \,|\, j \to i,\, i \in \alpha, j \in \beta] \\
c_{\mu\sigma}^{\alpha\beta} &:= \text{Cov}[\, g^{\text{pre}}(\nu_j)\nu_j, g^{\text{pre}}(\nu_j)^2\nu_j \,|\, j \to i,\, i \in \alpha, j \in \beta].
\end{aligned} \tag{S107}$$

The set of mean-field parameters to be determined is then $\boldsymbol{\theta} := (m_\mu^{\alpha\beta}, s_\mu^{2,\alpha\beta}, m_\sigma^{\alpha\beta}, s_\sigma^{2,\alpha\beta}, c_{\mu\sigma}^{\alpha\beta})_{\alpha,\beta\in\{E,I\}}$. The firing rate ν_i of a neuron $i \in \alpha$ is again determined by $\boldsymbol{\theta}$ and by a set of identity variables associated to that neuron, $\boldsymbol{X}_i^\alpha = (K_i^E, Y_i^{\alpha E}, Z_i^{\alpha E}, K_i^I, Y_i^{\alpha I}, Z_i^{\alpha I})$, whose distribution also depends on the mean-field parameters. To compute ν_i from $\boldsymbol{\theta}$ and $\boldsymbol{X}_i^\alpha$ we must solve a one-dimensional equation on ν_i:

$$\nu_i = \phi\left(\mu_\alpha\left(\nu_i, \boldsymbol{\theta}, \boldsymbol{X}_i^\alpha\right), \sigma_\alpha\left(\nu_i, \boldsymbol{\theta}, \boldsymbol{X}_i^\alpha\right)\right), \tag{S108a}$$

$$\begin{aligned}
\mu_\alpha\left(\nu_i, \boldsymbol{\theta}, \boldsymbol{X}_i^\alpha\right) &= \tau\left[g^{\text{post}}(\nu_i)\left(K_i^E\, m_\mu^{\alpha E} + \sqrt{K_i^E}Y_i^{\alpha E} - K_i^I\, m_\mu^{\alpha I} - \sqrt{K_i^I}Y_i^{\alpha I}\right) + K_{\text{ext}}w_{\text{ext}}\nu_{\text{ext}}\right] \\
\sigma^2_\alpha\left(\nu_i, \boldsymbol{\theta}, \boldsymbol{X}_i^\alpha\right) &= \tau\left[g^{\text{post}}(\nu_i)^2\left(K_i^E\, m_\sigma^{\alpha E} + \sqrt{K_i^E}Z_i^{\alpha E} + K_i^I\, m_\sigma^{\alpha I} + \sqrt{K_i^I}Z_i^{\alpha I}\right) + K_{\text{ext}}w^2_{\text{ext}}\nu_{\text{ext}}\right].
\end{aligned} \tag{S108b}$$

We denote by $\Phi_\alpha = \Phi_\alpha(\boldsymbol{\theta}, \boldsymbol{X}_i^\alpha)$ a mapping that, given $\boldsymbol{\theta}$ and $\boldsymbol{X}_i^\alpha$, returns a solution to Eq. (S108) on ν_i. Again, the variables K_i^E, K_i^I are distributed according to the excitatory and inhibitory in-degree distribution imposed in the network and $(Y_i^{\alpha E}, Z_i^{\alpha E})$, $(Y_i^{\alpha I}, Z_i^{\alpha I})$ are normal bivariate independent vectors with zero mean and covariance matrix $\boldsymbol{\Sigma}^{\alpha E} = \boldsymbol{\Sigma}^{\alpha E}(\boldsymbol{\theta})$ and $\boldsymbol{\Sigma}^{\alpha I} = \boldsymbol{\Sigma}^{\alpha I}(\boldsymbol{\theta})$ [see Eqs. (S105), (S106), (S107)]. For all $\beta \in \{E, I\}$, the vector $(Y_i^{\alpha\beta}, Z_i^{\alpha,\beta})$ is independent of K_i^E and K_i^I, and the identity vectors of all the neurons within population α, $\boldsymbol{X}_1^\alpha, \cdots, \boldsymbol{X}_{N_\alpha}^\alpha$, are i.i.d.

The firing rate distribution can thus be reconstructed once the mean-field parameter set $\boldsymbol{\theta}$ is known. By definition,

$$\begin{aligned}
m_\mu^{\alpha\beta} &= \int_0^\infty\int_{-\infty}^\infty\int_{-\infty}^\infty\int_0^\infty\int_{-\infty}^\infty\int_{-\infty}^\infty g^{\mathrm{pre}}\left(\Phi_\beta(\boldsymbol{\theta},\boldsymbol{x})\right)\,\Phi_\beta(\boldsymbol{\theta},\boldsymbol{x})\,\rho_{\boldsymbol{X}}^{\boldsymbol{\theta},\alpha\beta}(\boldsymbol{x})\,\mathrm{d}\boldsymbol{x} &&=: G_{m_\mu}^{\alpha\beta}(\boldsymbol{\theta})\\
s_\mu^{2,\alpha\beta} &= \int_0^\infty\int_{-\infty}^\infty\int_{-\infty}^\infty\int_0^\infty\int_{-\infty}^\infty\int_{-\infty}^\infty \left[g^{\mathrm{pre}}\left(\Phi_\beta(\boldsymbol{\theta},\boldsymbol{x})\right)\,\Phi_\beta(\boldsymbol{\theta},\boldsymbol{x}) - m_\mu^{\alpha\beta}\right]^2\rho_{\boldsymbol{X}}^{\boldsymbol{\theta},\alpha\beta}(\boldsymbol{x})\,\mathrm{d}\boldsymbol{x} &&=: G_{s_\mu^2}^{\alpha\beta}(\boldsymbol{\theta})\\
m_\sigma^{\alpha\beta} &= \int_0^\infty\int_{-\infty}^\infty\int_{-\infty}^\infty\int_0^\infty\int_{-\infty}^\infty\int_{-\infty}^\infty g^{\mathrm{pre}}\left(\Phi_\beta(\boldsymbol{\theta},\boldsymbol{x})\right)^2\,\Phi_\beta(\boldsymbol{\theta},\boldsymbol{x})\,\rho_{\boldsymbol{X}}^{\boldsymbol{\theta},\alpha\beta}(\boldsymbol{x})\,\mathrm{d}\boldsymbol{x} &&=: G_{m_\sigma}^{\alpha\beta}(\boldsymbol{\theta})\\
s_\sigma^{2,\alpha\beta} &= \int_0^\infty\int_{-\infty}^\infty\int_{-\infty}^\infty\int_0^\infty\int_{-\infty}^\infty\int_{-\infty}^\infty \left[g^{\mathrm{pre}}\left(\Phi_\beta(\boldsymbol{\theta},\boldsymbol{x})\right)^2\,\Phi_\beta(\boldsymbol{\theta},\boldsymbol{x}) - m_\sigma^{\alpha\beta}\right]^2\rho_{\boldsymbol{X}}^{\boldsymbol{\theta},\alpha\beta}(\boldsymbol{x})\,\mathrm{d}\boldsymbol{x} &&=: G_{s_\sigma^2}^{\alpha\beta}(\boldsymbol{\theta})\\
c_{\mu\sigma}^{\alpha\beta} &= \int_0^\infty\int_{-\infty}^\infty\int_{-\infty}^\infty\int_0^\infty\int_{-\infty}^\infty\int_{-\infty}^\infty \left[g^{\mathrm{pre}}\left(\Phi_\beta(\boldsymbol{\theta},\boldsymbol{x})\right)\,\Phi_\beta(\boldsymbol{\theta},\boldsymbol{x}) - m_\mu^{\alpha\beta}\right]\left[g^{\mathrm{pre}}\left(\Phi_\beta(\boldsymbol{\theta},\boldsymbol{x})\right)^2\,\Phi_\beta(\boldsymbol{\theta},\boldsymbol{x}) - m_\sigma^{\alpha\beta}\right]\rho_{\boldsymbol{X}}^{\boldsymbol{\theta},\alpha\beta}(\boldsymbol{x})\,\mathrm{d}\boldsymbol{x} &&=: G_{c_{\mu\sigma}}^{\alpha\beta}(\boldsymbol{\theta}),
\end{aligned}\tag{S109}$$

$\alpha,\beta\in\{E,I\}$, where $\boldsymbol{x}=(k^E,y^E,z^E,k^I,y^I,z^I)$ and $\rho_{\boldsymbol{X}}^{\boldsymbol{\theta},\alpha\beta}(\boldsymbol{x})$ is the p.d.f. of $\boldsymbol{X}_i^\beta=(K_i^E,Y_i^{\beta E},Z_i^{\beta E},K_i^I,Y_i^{\beta I},Z_i^{\beta I})$ for a neuron $i\in\beta$ that is presynaptic to a neuron in α:

$$\rho_{\boldsymbol{X}}^{\boldsymbol{\theta},\alpha\beta}(\boldsymbol{x}) = \rho_K^{\mathrm{pre},\alpha}(k^\alpha)\,\rho_K^{\bar\alpha}(k^{\bar\alpha})\,\rho_{Y,Z}^{\boldsymbol{\theta},\beta E}(y^E,z^E)\,\rho_{Y,Z}^{\boldsymbol{\theta},\beta I}(y^I,z^I),\tag{S110}$$

with

$$\bar\alpha = \begin{cases} I & \text{if } \alpha = E\\ E & \text{if } \alpha = I\end{cases}\tag{S111}$$

and $\rho_K^{\mathrm{pre},\alpha}$ being the p.d.f. of the in-degree from population α of a neuron that is presynaptic to a neuron in α (see section 4 for details), ρ_K^α being the p.d.f. of the in-degree from population α of a random neuron, $\rho_{Y,Z}^{\boldsymbol{\theta},\beta\gamma}$ being the p.d.f. of a normal bivariate vector with mean $\mathbf{0}$ and covariance matrix $\boldsymbol{\Sigma}^{\beta\gamma}(\boldsymbol{\theta})$, $\gamma\in\{E,I\}$.

The mean-field parameter set $\boldsymbol{\theta}$ is thus found by solving the system of 20 unknowns and 20 equations

$$\boldsymbol{\theta} = G(\boldsymbol{\theta}),\tag{S112}$$

with $G(\boldsymbol{\theta}) := \left(G_{m_\mu}^{\alpha\beta} G_{s_\mu^2}^{\alpha\beta}, G_{m_\sigma}^{\alpha\beta}, G_{s_\sigma^2}^{\alpha\beta}, G_{c_{\mu\sigma}}^{\alpha\beta}\right)_{\alpha,\beta\in\{E,I\}}(\boldsymbol{\theta})$ and the component functions are defined in Eq. (S109).

As in the non-plastic network, the absence of degree correlations greatly simplifies the mean-field equations: the moments of Eq. (S107) become independent of the condition and of both α and β, so the mean-field parameter set has only 5 parameters: $\boldsymbol{\theta}=(m_\mu,s_\mu^2,m_\sigma,s_\sigma^2,c_{\mu\sigma})$, with

$$\begin{aligned}
m_\mu &:= \mathbb{E}[\,g^{\mathrm{pre}}(\nu_j)\nu_j]\\
s_\mu &:= \mathrm{Var}[\,g^{\mathrm{pre}}(\nu_j)\nu_j]\\
m_\sigma &:= \mathbb{E}[\,g^{\mathrm{pre}}(\nu_j)^2\nu_j]\\
s_\sigma &:= \mathrm{Var}[\,g^{\mathrm{pre}}(\nu_j)^2\nu_j]\\
c_{\mu\sigma} &:= \mathrm{Cov}[\,g^{\mathrm{pre}}(\nu_j)\nu_j,\,g^{\mathrm{pre}}(\nu_j)^2\nu_j],
\end{aligned}\tag{S113}$$

where j is a random neuron in the network. The firing rate of a neuron i with identity variables $\boldsymbol{X}_i=(K_i^E,Y_i^E,Z_i^E,K_i^I,Y_i^I,Z_i^I)$ is thus

$$\nu_i = \phi\left(\mu\left(\nu_i,\boldsymbol{\theta},\boldsymbol{X}_i\right),\sigma\left(\nu_i,\boldsymbol{\theta},\boldsymbol{X}_i\right)\right),\tag{S114a}$$

$$\begin{aligned}
\mu\left(\nu_i,\boldsymbol{\theta},\boldsymbol{X}_i\right) &= \tau\left[g^{\mathrm{post}}(\nu_i)\left(K_i^E\,m_\mu + \sqrt{K_i^E}Y_i^E - K_i^I\,m_\mu - \sqrt{K_i^I}Y_i^I\right) + K_{\mathrm{ext}}w_{\mathrm{ext}}\nu_{\mathrm{ext}}\right]\\
\sigma^2\left(\nu_i,\boldsymbol{\theta},\boldsymbol{X}_i\right) &= \tau\left[g^{\mathrm{post}}(\nu_i)^2\left(K_i^E\,m_\sigma + \sqrt{K_i^E}Z_i^E + K_i^I\,m_\sigma + \sqrt{K_i^I}Z_i^I\right) + K_{\mathrm{ext}}w_{\mathrm{ext}}^2\nu_{\mathrm{ext}}\right].
\end{aligned}\tag{S114b}$$

If $\Phi=\Phi(\boldsymbol{\theta},\boldsymbol{X}_i)$ is a mapping that, given $\boldsymbol{\theta}$ and $\boldsymbol{X}_i$, returns a solution to Eq. (S114) on ν_i, then the mean-field parameters fulfill

$$\begin{aligned}
m_\mu &= \int_0^\infty\int_{-\infty}^\infty\int_{-\infty}^\infty\int_0^\infty\int_{-\infty}^\infty\int_{-\infty}^\infty g^{\mathrm{pre}}\left(\Phi(\boldsymbol{\theta},\boldsymbol{x})\right)\,\Phi(\boldsymbol{\theta},\boldsymbol{x})\,\rho_{\boldsymbol{X}}^{\boldsymbol{\theta}}(\boldsymbol{x})\,\mathrm{d}\boldsymbol{x} &&=: G_{m_\mu}(\boldsymbol{\theta})\\
s_\mu^2 &= \int_0^\infty\int_{-\infty}^\infty\int_{-\infty}^\infty\int_0^\infty\int_{-\infty}^\infty\int_{-\infty}^\infty \left[g^{\mathrm{pre}}\left(\Phi(\boldsymbol{\theta},\boldsymbol{x})\right)\,\Phi(\boldsymbol{\theta},\boldsymbol{x}) - m_\mu\right]^2\rho_{\boldsymbol{X}}^{\boldsymbol{\theta}}(\boldsymbol{x})\,\mathrm{d}\boldsymbol{x} &&=: G_{s_\mu^2}(\boldsymbol{\theta})\\
m_\sigma &= \int_0^\infty\int_{-\infty}^\infty\int_{-\infty}^\infty\int_0^\infty\int_{-\infty}^\infty\int_{-\infty}^\infty g^{\mathrm{pre}}\left(\Phi(\boldsymbol{\theta},\boldsymbol{x})\right)^2\,\Phi(\boldsymbol{\theta},\boldsymbol{x})\,\rho_{\boldsymbol{X}}^{\boldsymbol{\theta}}(\boldsymbol{x})\,\mathrm{d}\boldsymbol{x} &&=: G_{m_\sigma}(\boldsymbol{\theta})\\
s_\sigma^2 &= \int_0^\infty\int_{-\infty}^\infty\int_{-\infty}^\infty\int_0^\infty\int_{-\infty}^\infty\int_{-\infty}^\infty \left[g^{\mathrm{pre}}\left(\Phi(\boldsymbol{\theta},\boldsymbol{x})\right)^2\,\Phi(\boldsymbol{\theta},\boldsymbol{x}) - m_\sigma\right]^2\rho_{\boldsymbol{X}}^{\boldsymbol{\theta}}(\boldsymbol{x})\,\mathrm{d}\boldsymbol{x} &&=: G_{s_\sigma^2}(\boldsymbol{\theta})\\
c_{\mu\sigma} &= \int_0^\infty\int_{-\infty}^\infty\int_{-\infty}^\infty\int_0^\infty\int_{-\infty}^\infty\int_{-\infty}^\infty \left[g^{\mathrm{pre}}\left(\Phi(\boldsymbol{\theta},\boldsymbol{x})\right)\,\Phi(\boldsymbol{\theta},\boldsymbol{x}) - m_\mu\right]\left[g^{\mathrm{pre}}\left(\Phi(\boldsymbol{\theta},\boldsymbol{x})\right)^2\,\Phi(\boldsymbol{\theta},\boldsymbol{x}) - m_\sigma\right]\rho_{\boldsymbol{X}}^{\boldsymbol{\theta}}(\boldsymbol{x})\,\mathrm{d}\boldsymbol{x} &&=: G_{c_{\mu\sigma}}(\boldsymbol{\theta}),
\end{aligned}\tag{S115}$$

where $\boldsymbol{x} = (k^E, y^E, z^E, k^I, y^I, z^I)$ and $\rho^{\boldsymbol{\theta}}_{\boldsymbol{X}}(\boldsymbol{x})$ is the p.d.f. of $\boldsymbol{X}_i = (K_i^E, Y_i^E, Z_i^E, K_i^I, Y_i^I, Z_i^I)$ for a random neuron i:

$$\rho^{\boldsymbol{\theta}}_{\boldsymbol{X}}(\boldsymbol{x}) = \rho_K^E(k^E)\,\rho_K^I(k^I)\,\rho^{\boldsymbol{\theta}}_{Y,Z}(y^E, z^E)\,\rho^{\boldsymbol{\theta}}_{Y,Z}(y^I, z^I), \tag{S116}$$

with ρ_K^α being the p.d.f. of the in-degree from population α of a random neuron, $\rho^{\boldsymbol{\theta}}_{Y,Z}$ being the p.d.f. of a normal bivariate vector with mean $\mathbf{0}$ and covariance matrix $\boldsymbol{\Sigma}(\boldsymbol{\theta})$.

Let us go back to the general scenario in which degrees might be correlated. Once system (S112) is solved and we know the value of $\boldsymbol{\theta}$, the synaptic weight of a randomly chosen connection from $j \in \beta$ to $i \in \alpha$ is computed as follows. If i and j have identity variables $\boldsymbol{X}_i^\alpha$ and $\boldsymbol{X}_j^\beta$, the firing rates of i and j are

$$\nu_i = \Phi_\alpha(\boldsymbol{\theta}, \boldsymbol{X}_i^\alpha), \qquad \nu_j = \Phi_\beta(\boldsymbol{\theta}, \boldsymbol{X}_j^\beta) \tag{S117}$$

and the synaptic weight of the connection $i \leftarrow j$ is given by Eq. (S100). The in-degrees of the neurons involved in the connection, K_i^γ and K_j^γ, $\gamma \in \{E, I\}$, do not necessarily follow the in-degree distribution imposed in the network. Knowing that $j \to i$, $i \in \alpha$, $j \in \beta$ always biases the in-degree of i from population β and can bias (if degree correlations are present) the in-degree of j from population α (see section 4 for details).

REFERENCES

1. L. M. Ricciardi. *Lecture Notes in Biomathematics | Diffusion Processes and Related Topics in Biology*, volume 14. Springer-Verlag, 1st edition, 1977. ISBN 978-3-642-93059-1.
2. J. Feng, editor. *Computational Neuroscience: A Comprehensive Approach*. Chapman & Hall/CRC, 2004. ISBN 1-58488-362-6.
